# Five Novel Freshwater Ascomycetes Indicate High Undiscovered Diversity in Lotic Habitats in Thailand

**DOI:** 10.3390/jof7020117

**Published:** 2021-02-05

**Authors:** Mark S. Calabon, E. B. Gareth Jones, Saranyaphat Boonmee, Mingkwan Doilom, Saisamorn Lumyong, Kevin D. Hyde

**Affiliations:** 1 Center of Excellence in Fungal Research, Mae Fah Luang University, Chiang Rai 57100, Thailand; mscalabon@up.edu.ph (M.S.C.); saranyaphat.boo@mfu.ac.th (S.B.); 2 School of Science, Mae Fah Luang University, Chiang Rai 57100, Thailand; 3 Department of Botany and Microbiology, College of Science, King Saud University, P.O Box 2455, Riyadh 11451, Saudi Arabia; torperadgj@gmail.com; 4 Department of Biology, Faculty of Science, Chiang Mai University, Chiang Mai 50200, Thailand; j_hammochi@hotmail.com (M.D.); scboi009@gmail.com (S.L.); 5 Research Center of Microbial Diversity and Sustainable Utilization, Chiang Mai University, Chiang Mai 50200, Thailand; 6 CAS Key Laboratory for Plant Diversity and Biogeography of East Asia, Kunming Institute of Botany, Chinese Academy of Sciences, Kunming 650201, China; 7 Innovative Institute for Plant Health, Zhongkai University of Agriculture and Engineering, Guangzhou 510225, China; 8 Academy of Science, The Royal Society of Thailand, Bangkok 10300, Thailand

**Keywords:** 6 new taxa, aquatic fungi, Dothideomycetes, freshwater fungi, multi-loci phylogenetic analyses, *Neoxylomyces*, Sordariomycetes, tropical mycology

## Abstract

An investigation of freshwater fungi in Thailand resulted in the collection of one new monotypic genus, *Neoxylomyces*, and a novel species each in *Camposporium*, *Brunneofusispora*, *Rattania*, *Neoxylomyces*, and *Phaeoacremonium*. *Camposporium dulciaquae* resembles *C. septatum* in conidial morphology and number of septa but differs in conidial sizes. *Brunneofusispora hyalina* is similar to *B. sinensis* in conidiogenesis and conidial shape but differs in the sizes of conidiomata and conidiogenous cells. *Rattania aquatica* is the second species in *Rattania*, while *Phaeoacremonium thailandense* is the third species recorded from freshwater habitats. A new genus, *Neoxylomyces*, typified by *N. multiseptatus*, is similar to *Xylomyces giganteus*, but differs in the number of septa, chlamydospore measurements, and absence of a mucilaginous coating around the chlamydospores. These novel taxa form an independent lineage distinct from other species based on multi-loci phylogenetic analyses. Descriptions, illustrations, and notes are provided for each taxon. These new freshwater ascomycetes add to the increasing number of fungi known from Thailand and it is now evident that there are numerous novel taxa awaiting to be described as new freshwater habitats are explored. An update of newly discovered taxa in the widely studied freshwater habitats of Thailand over the last five years is also provided.

## 1. Introduction

Freshwater fungi are a diverse and heterogenous taxonomic group occurring on a wide variety of substrates and with a global distribution [1,2,3,4]. Jones et al. [2] estimated the number of freshwater fungi to be between 3069–4145, which is lower as compared to marine fungi with an estimated 12,500 species [5,6]. There are 1898 species under 767 genera of marine fungi listed in www.marinefungi.org (accessed on 13 January 2021) [6], but there are no recent published reports of freshwater fungal numbers [2,7]. An online platform on freshwater fungi, www.freshwarterfungi.org (accessed on 13 January 2021). is presently being compiled. This comprehensive database presently includes 451 species of freshwater Sordariomycetes, while the outline of freshwater Dothideomycetes is in preparation [8]. Many habitats and substrates are still not well-explored and freshwater fungi are likely to be more numerous than those thriving in marine environments.

Freshwater fungi have been relatively well-studied in Asia over the past decade, particularly in China and Thailand. Several new taxa have been introduced and existing taxa lacking molecular data have been recollected and sequenced resolving their taxonomic placements [8,9,10,11,12,13]. Several monographs and taxonomic revisions of freshwater fungi have also been published [10,14,15,16]. In Thailand, Zhang et al. [17] listed 173 species belonging to 112 genera recorded from freshwater habitats in 2010. Hu et al. [18] reported 782 species of freshwater fungi in China. Since then, many additional novel taxa have been described in these countries, bringing the number even higher (see [9,10,11,14,19,20,21,22,23,24,25,26]. In this paper, six novel taxa of freshwater ascomycetes in Thailand are introduced by combining multi-loci phylogeny and morphology approaches. It appears that there are numerous new taxa awaiting to be discovered and described as new freshwater habitats are explored, or a particular genus is studied with molecular data, wherein its diversity is much higher than previously anticipated [10,14,25,27].

We are carrying out surveys of freshwater fungi on submerged wood in streams along a north–south gradient in the Asia region [28] and here we introduce one new genus, *Neoxylomyces* (Phyllachorales genera *incertae sedis*, Phyllachorales), and one new species in each of *Camposporium* (Melanommataceae, Pleosporales), *Brunneofusispora* (Occultibambusaceae, Pleosporales), *Rattania* (Chaetosphaeriaceae, Chaetosphaeriales), and *Phaeoacremonium* (Togniniaceae, Togniniales).

## 2. Materials and Methods

### 2.1. Sample Collection, Morphological Observation, and Fungal Isolation

Samples of submerged decayed wood were collected from a freshwater stream in Chiang Mai Province (19°07.200′ N, 98°44.044′ E) and river in Tak Province in Thailand (17°28′20.7834″ N, 98°1′16.3236″ E) and treated as detailed in Senanayake et al. [29]. The samples were incubated for five days and periodically observed using stereomicroscope to check the presence of fruiting bodies. Micromorphological features were photographed using a Motic SMZ 168 Series dissection microscope for fungal structures on the woody substrate while microscopic characters were documented using Nikon Eclipse 80i microscope-camera system. Tarosoft (R) Image FrameWork was used to measure the micromorphological characters and photographic plates’ preparation was done using Adobe Photoshop CC 2020. The mean size/length of at least 10 ascomata/conidiomata, 20 conidiophores, 20 asci, and 30 conidia/ascospores were measured and recorded. Single-spore isolation was used to obtain pure cultures and colonial characteristics in malt extract agar (MEA) were described. Herbarium type specimens were deposited in Mae Fah Luang University (MFLU). Ex-type living cultures were deposited at Mae Fah Luang University Culture Collection (MFLUCC). The new species were registered in Faces of Fungi (http://http://www.facesoffungi.org/ (accessed on 13 January 2021)) [30] and MycoBank databases (https://www.mycobank.org/ (accessed on 13 January 2021)) [31]

### 2.2. DNA Extraction, PCR Amplification, and Sequencing

DNA extraction, polymerase chain reaction (PCR) amplification, agarose gel electrophoresis, PCR product purification, and sequencing were carried out as detailed in Dissayanake et al. [32] with the following modifications. Fungal mycelia from pure cultures grown in malt extract agar (MEA) (Difco™) for 30 days were scraped using a sterilized scalpel and kept in a 1.5 mL microcentrifuge tube. Genomic DNA was extracted using a Biospin Fungus Genomic DNA Extraction Kit (BioFlux^®^, Hangzhou, China) following the manufacturer’s protocol. Polymerase chain reaction (PCR) was used to amplify six markers: the nuclear ribosomal large subunit 28S rRNA gene (LSU), the nuclear ribosomal small subunit 18S rRNA gene (SSU), nuclear ribosomal internal transcribed spacers (ITS), and fragments of the translation elongation factor 1-alpha (*TEF1-α*), β-tubulin (*TUB2*) and Actin (*ACT*) genes. LSU was amplified using the primers LROR and LR5 [33], while SSU was amplified using the primers NS1 and NS4 [34]. For ITS, primers ITS5 and ITS4 were used [34]. *TEF1-α* was amplified using primers EF1–983F and EF1–2218R [35]. Partial regions of the *TUB2* and *ACT* gene were amplified using the primer pairs T1 [36] and Bt2b [37], ACT-512F and ACT-783R [38], respectively. Polymerase chain reaction was performed in a volume of 25 μL, which contained 12.5 μL of 2× Power Taq PCR Master Mix (Bioteke Co., Jiangsu, China), 1 μL of each primer (10 μM), 1 μL genomic DNA, and 9.5 μL deionized water. The PCR thermal cycle programs for LSU, SSU, ITS and TEF1-α amplification were as follows: initial denaturing step of 94 °C for 3 min, followed by 40 cycles of denaturation at 94 °C for 45 s, annealing at 51 °C (*TUB2*) or 60 °C (ACT) or 55 °C (ITS, LSU, SSU, *TEF1-α*) for 50 sec, elongation at 72 °C for 1 min, and final extension at 72 °C for 10 min. Agarose gel electrophoresis was done to confirm the presence of amplicons at the expected molecular weight. PCR products were purified and sequenced with the primers mentioned above at a commercial sequencing provider (Beijing Qingke Biotechnology Co., Ltd., Beijing, China). A BLAST search of the newly generated sequences was carried out to exclude contamination and to search for related taxa in GenBank database (www.ncbi.nlm.nih.gov/blast/ (accessed on 13 January 2021)).

### 2.3. Phylogenetic Analyses

Multi-loci phylogenetic analysis followed Dissayanake et al. [32]. The taxa table was assembled based on the closest matches from the BLASTn search results and from recently published data. Sequences generated from each marker were analyzed along with other sequences retrieved from GenBank. The individual loci matrix was aligned with MAFFT v.7 using the web server (http://mafft.cbrc.jp/alignment/server (accessed on 13 January 2021); [39]) with the following settings: L-INS-i tree-based iterative refinement methods, 20PAM/k = 2 scoring matrix for nucleotide sequences and 1.53 gap opening penalty. Alignment was further refined manually, where necessary, using BioEdit v.7.0.9.0 [40]. Aligned sequences were automatically trimmed using TrimAl v. 1.3 on the web server (http://phylemon.bioinfo.cipf.es/utilities.html (accessed on 13 January 2021)) with the gappyout method. The online tool “ALTER” was used to convert the alignment file to phylip and nexus formats [41]. Phylogenetic analyses of both individual and combined gene data were performed using maximum likelihood (ML) and Bayesian inference (BI).

Maximum likelihood analysis was performed using RAxML-HPC2 on XSEDE on the CIPRES web portal [42,43,44] (http://www.phylo.org/portal2/ (accessed on 13 January 2021); [45]). The GTR + GAMMA model of nucleotide evolution was used. RAxML rapid bootstrapping of 1000 replicates was performed. The best-fit evolutionary models for individual and combined dataset were estimated under the Akaike Information Criterion (AIC) using jModeltest 2.1.10 on the CIPRES web portal and each resulted to GTR + I+G model [46]. Bayesian inference analyses was performed using MrBayes v. 3.2.6 on XSEDE at the CIPRES webportal [47]. The parameter setting of the Bayesian analysis is detailed on the phylogenetic trees of each species. Trees were sampled every 100 generations and all other parameters were left as default. Newly generated sequences were deposited in GenBank, and alignments and trees were deposited in TreeBASE (www.treebase.org (accessed on 13 January 2021)).

## 3. Results

### 3.1. Taxonomy

#### 3.1.1. Dothideomycetes O.E. Erikss. & Winka, Myconet 1(1): 5 (1997)

Dothideomycetes are characterized by bitunicate asci with fissitunicate dehiscence, and ascolocular ascomatal development [15,48]. The class comprises 38 orders and 211 families and is considered to be the largest and most ecologically diverse class of ascomycetes [49]. Two new taxa are introduced in this article.

Pleosporales Luttr. ex M.E. Barr, Prodr. Cl. Loculoasc. (Amherst): 67 (1987)

Wijayawardene et al. [49] listed 91 families and 566 genera under Pleosporales, with 48 genera in Pleosporales *incertae sedis*. It is the largest order of Dothideomycetes with members characterized mostly by flask-shaped pseudothecia [48]. In this paper, two new pleosporalean fungi are documented.

Melanommataceae G. Winter, Rabenhorst’s Kryptogamen-Flora, Pilze—Ascomyceten, Edn 2 1(2): 220 (1885)

Melanommataceae was introduced by Winter [50] and *Melanomma* was regarded as the type genus based on its diagnostic character of trabeculate pseudoparaphyses. The latest treatment of the family by Hongsanan et al. [15] and Wijayawardene et al. [49] with 33 accepted genera in Melanommataceae are followed here.

*Camposporium* Harkn., Bull. South. Calif. Acad. Sci. 1: 37 (1884)

*Camposporium* was introduced by Harkness [51], with the single species *C. antennatum*. *Camposporium* is characterized by dematiaceous conidiophores, terminal, integrated, denticulate conidiogenous cells, and cylindrical and elongate, multiseptate conidia with one or more cylindrical appendages at the apex [52,53,54,55]. Twenty species are accepted in this genus (Species Fungorum 2020, http://www.speciesfungorum.org/Names/Names.asp (accessed on 13 January 2021)) and we introduced one novel *Camposporium* species in this paper.

**Figure 1:**
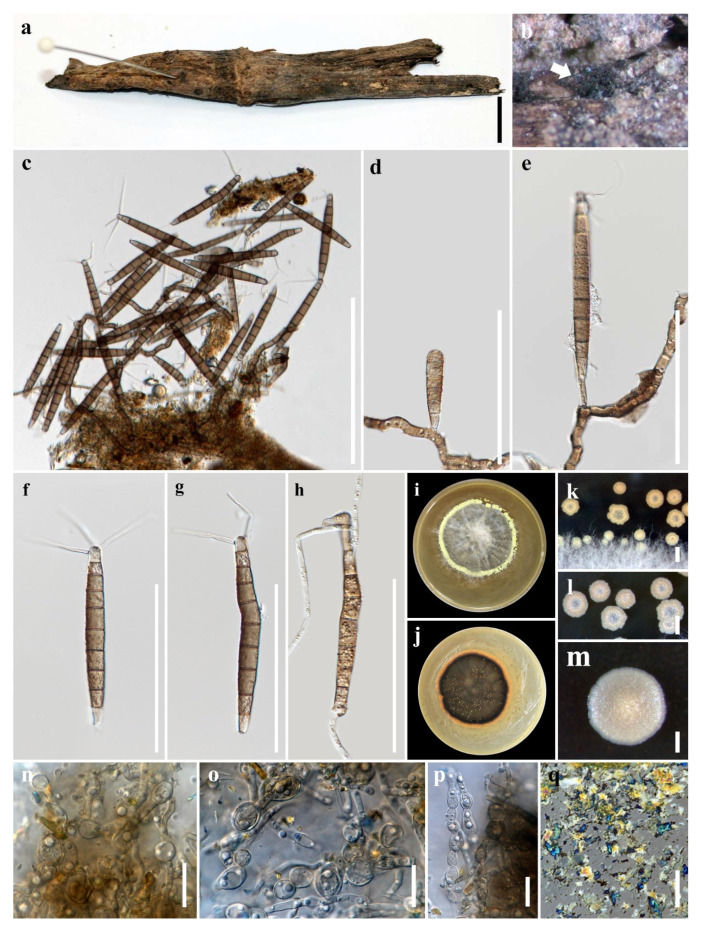
*Camposporium dulciaquae* (MFLU 21–0015, holotype). (**a**) Substrate; (**b**) colonies on wood; (**c**–**e**) conidiophores with conidia; (**f**,**g**) conidia; (**h**) germinating conidium; (**i**,**j**) culture on malt extract agar (MEA) from surface and reverse; (**k**–**m**) crystals in culture; (**n**–**p**) chlamydospores; (**q**) crystal structure observed in microscope. Scale bars: (**a**,**m**) 10 mm; (**c**) 200 μm; (**d**–**h**) 100 μm; (**k**,**l**) 50 μm; (**n**–**q**) 20 μm.

*Camposporium dulciaquae* M.S. Calabon & K.D. Hyde, sp. nov. (Figure 1)

MycoBank number: MB838551; Facesoffungi number: FoF 09156

Etymology: of freshwater

Holotype: MFLU 21–0015

Saprobic on submerged decaying wood in freshwater. **Sexual morph**: Undetermined. **Asexual morph**: Hyphomycetous. *Colonies* on natural substrate, effuse, golden brown, velvety. *Mycelium* mostly immersed, composed of white, septate, branched and guttulate hyphae. *Conidiophores* 16–95 × 5–9 μm ($\bar{x}$ = 54.4 × 6.7 μm, *n* = 20) macronematous, mononematous, often procumbent on substrate, light brown to brown, unbranched, irregularly cylindrical, flexuous, septate, thick-walled. *Conidiogenous cells* 9–41 × 3–5 μm ($\bar{x}$ = 22.2 × 4.4 μm, *n* = 20) monoblastic, terminal, integrated, subcylindrical, pale brown. *Conidia* 100–130 μm ($\bar{x}$ = 115 μm, *n* = 30) long, 8.5–13 μm ($\bar{x}$ = 10.7 μm, *n* = 30) wide at middle, 4–9 μm ($\bar{x}$ = 6.1 μm, *n* = 30) wide at base, solitary, dry, cylindrical, elongate, median brown, paler at base, finely verrucose, 8–11-septate, not constricted or slightly constricted at septa, apex rounded, basal cell truncate, apical cell gives rise to (2–)3 simple appendages; *appendage* hyaline, aseptate, smooth, tapering from base to apex. *Chlamydospores* 8–18 × 5–13 μm ($\bar{x}$ = 12.4–9.1 μm, *n* = 30) diameter, numerous, mostly in chains, intercalary or solitary, globose to subglobose, hyaline.

Culture characteristics: Conidia germinating on malt extract agar (MEA) within 24 h. Germ tubes produced from the basal and apical cell of conidia. Colonies growing on MEA, reaching 20 mm in 2 weeks at 25 °C. Mycelia superficial, circular, with entire edge, flat, rugose, from above mossy gray in the center and pale yellow at the edge, from below brown at the center then becoming yellow orange at the edge. Chlamydospores produced in culture and induced with plant tissues within 60 days. Formation of crystals in the culture was observed.

Material examined: THAILAND, Chiang Mai Province, Mushroom Research Center, on decaying wood submerged in a freshwater stream, 29 January 2019, S. Boonmee, SB14-7 (MFLU 21–0015, holotype), ex-type living culture, MFLUCC 21–0009.

GenBank accession numbers: LSU = MT860430, SSU = MW485612, ITS = MT864352, *TEF1-α* = MW537104

**Figure 2:**
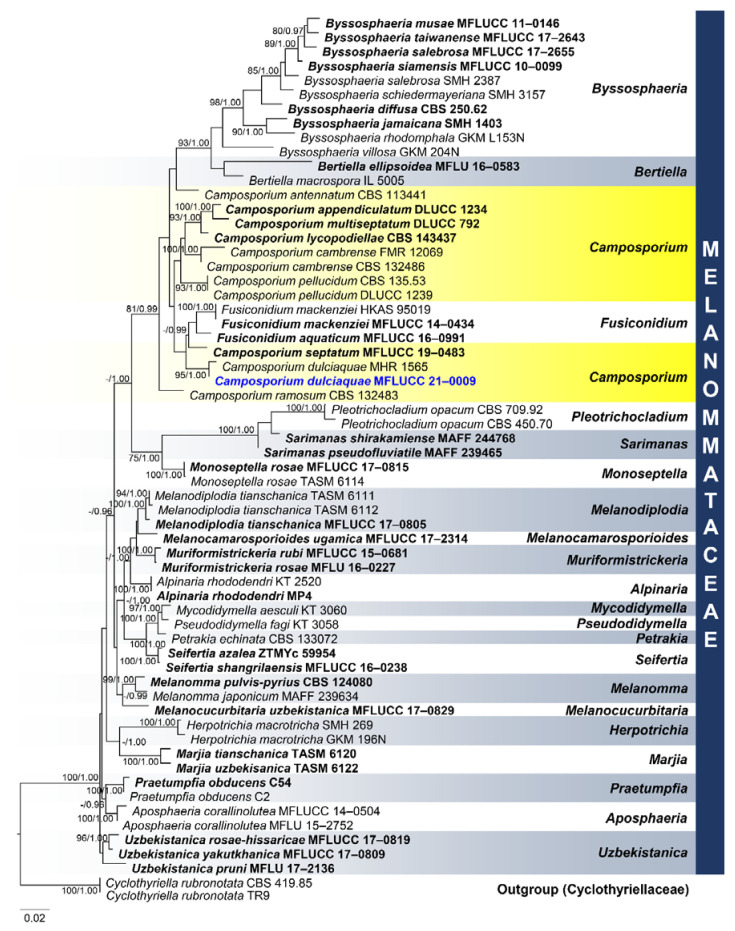
Phylogram generated from maximum likelihood analysis based on combined large subunit (LSU), small subunit (SSU), internal transcribed spacers (ITS), and *TEF1-α* sequence data representing the species of Melanommataceae. Related sequences are taken from Hyde et al. [57]. Sixty-two taxa were included in the combined analyses, which comprised 3433 characters (LSU = 982 bp, SSU = 963 bp, ITS = 545 bp, *TEF1-α* = 943) after alignment. The best scoring RAxML tree with a final likelihood value of -18023.445951 is presented. The matrix had 1115 distinct alignment patterns, with 44.01% of undetermined characters or gaps. Estimated base frequencies were as follows: A = 0.244338, C = 0.242233, G = 0.271538, T = 0.241891; substitution rates: AC = 1.712430, AG = 2.882169, AT = 1.678717, CG = 0.989358, CT = 10.389868, GT = 1.000000; gamma distribution shape parameter α = 0.154096. Bootstrap support values for ML equal to or greater than 75% and BYPP equal to or greater than 0.95 are given above the nodes. *Cyclothyriella rubronotata* (CBS 419.85; TR9) in Cyclothyriellaceae were used as the outgroup taxa. The newly generated sequence is indicated in blue. The ex-type strains are indicated in bold.

Notes: *Camposporium dulciaquae* closely resembles *Camposporium septatum* based on conidial morphology with 2–3 appendages and number of septa, but the former has larger conidia (100–130 μm long, 8.5–13 μm wide at middle, 4–9 μm wide at base versus 98–125 μm long, 7–11.5 μm wide at middle, 3.5–6 μm wide at base). BLAST results of ITS and *TEF1-α* sequence data were *C. cambrense* CBS 132,486 (95% similarity) and *C. septatum* MFLUCC 19–0483 MFLUCC 16–0274 (97% similarity), respectively. Phylogenetic analyses of the combined LSU, SSU, ITS, and *TEF1-α* sequence dataset showed that *C. dulciaquae* clustered with *C. septatum* (MFLUCC 19–0483), *Fusiconidium aquaticum* (MFLUCC 16–0991 and *F. mackenziei* (HKAS 95019; MFLUCC 14-0434) (Figure 2). [27]. In addition, an unknown species of *Camposporium* (MHR 1565) isolated from a dead wood in Nan Province, Thailand grouped as a sister taxon to *C. dulciaquae* with high bootstrap support (95% ML, 1.00 BYPP) [56]. The former did not have morphological data, but since comparison of its LSU sequence with the latter revealed 2 base pair differences (0.25%, 794 bp), we considered this as another strain of *Camposporium dulciaquae*. A comparison of ITS and *TEF1-α* sequence data of *C. dulciaquae* differed by 19 (3.81%, 499 bp) and 33 (3.48%, 947 bp) base pairs with *C. septatum*, respectively. *Camposporium dulciaquae* had 33 (914 bp, 3.61%) and 32 (838 bp, 3.82%) base pair differences with *F. aquaticum* and *F. mackenziei* in *TEF1-α* region, respectively. *Camposporium dulciaquae* differed from *Fusiconidium* in conidiogenesis (monoblastic versus enteroblastic), conidial shape (cylindrical versus fusiform to ellipsoidal), and presence of apical appendage. Furthermore, *C. dulciaquae* fit to the description of the genus, so in this paper, we introduced a new species under *Camposporium*. A key to freshwater *Camposporium* is provided below: 
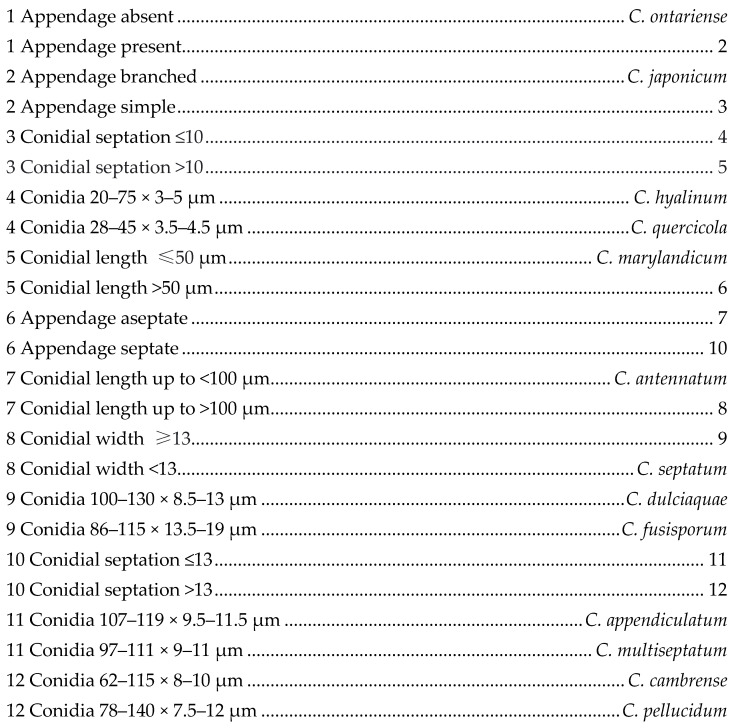


##### Occultibambusaceae D.Q. Dai & K.D. Hyde

Dai et al. [58] introduced Occultibambusaceae to accommodate *Neooccultibambusa, Occultibambusa, Seriascoma* and *Versicolorisporium*. *Brunneofusispora*, typified by *Brunneofusispora sinensis*, was introduced by Phookamsak et al. [59] as a new member of the family. The latest treatment of the family follows Hongsanan et al. [15] and Wijayawardene et al. [49] with 5 accepted genera in Occultibambusaceae.

*Brunneofusispora* S.K. Huang & K.D. Hyde, Fungal Diversity 95: 36 (2019)

*Brunneofusispora,* typified by *B. sinensis*, was introduced by Phookamsak et al. [59]. Hongsanan et al. [15] discussed the key differences of *Brunneofusispora* to other genera in Occultibambusaceae and Wanasinghe et al. [60] observed the coelomycetous asexual morph of *B. sinensis* and amended the generic and species description. Two species, *B. sinensis* and *B. clematidis*, have molecular data and are accepted in this genus (Species Fungorum 2020; http://www.speciesfungorum.org/Names/Names.asp (accessed on 13 January 2021)). *Brunneofusispora sinensis* was described from undetermined terrestrial wood near in a river [59] and *Magnolia denudata* [60] in China, while *B. clematidis* was observed in *Clematis subumbellata* by Phukhamsakda et al. [61]. In this paper, one novel coelomycetous *Brunneofusispora* species from a freshwater habitat is introduced.

**Figure 3:**
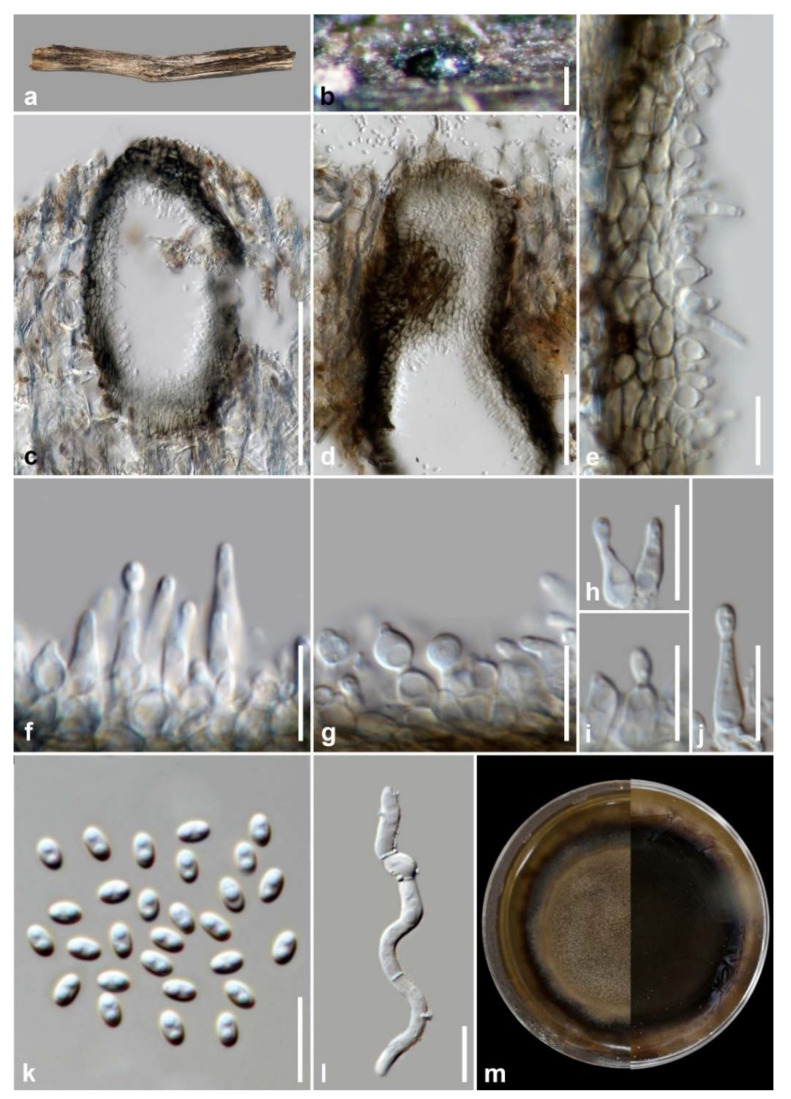
*Brunneofusispora hyalina* (MFLU 21–0016, holotype). (**a**) Host; (**b**) black conidiomata on the host; (**c**,**d**) vertical section of conidiomata; (**e**) vertical section of conidiomatal wall.; (**f**–**j**) conidiogenous cells and developing conidia; (**k**) conidia; (**l**) germinated conidium; (**m**) culture on MEA. Scale bars: (**b**,**c**)  100 µm; (**d**) 50 µm; (**e**–**l**) 10 µm.

*Brunneofusispora hyalina* M.S. Calabon & K.D. Hyde, sp. nov. (Figure 3)

MycoBank number: MB838552; Facesoffungi number: FoF 09531

Etymology: refers to hyaline conidia

Holotype: MFLU 21–0016

Saprobic on decaying wood submerged in freshwater habitats. **Sexual morph**: Undetermined. **Asexual morph**: *Mycelium* mostly immersed, composed of septate, branched, hyphae. *Conidiomata* 260–435 × 110–205 μm ($\bar{x}$ = 347 × 175 μm, *n* = 10), black, pycnidial, solitary, immersed to semi-immersed, globose to subglobose, ostiolate. *Ostiole* circular, papillate, laterally or centrically located. *Conidiomatal wall* 10–20 μm wide ($\bar{x}$ = 16 μm, *n* = 15), composed of thick-walled, dark brown to hyaline cells of *textura angularis*. *Conidiophores* reduced to conidiogenous cells. *Conidiogenous cells* 5–11 μm × 2–4 μm ($\bar{x}$ = 8 μm, *n* = 20), hyaline, thin-walled, enteroblastic, phialidic, smooth, cylindrical, subcylindrical, ampulliform, pyriform, swollen at base, discrete, producing a single conidium at apex. *Conidia* 2–4 × 1.3–2.6 μm ($\bar{x}$ = 2.7 × 1.9 μm, n = 50), aseptate, oblong, cylindrical to ovoid, tapered to apices, hyaline, smooth-walled.

Culture characteristics: Conidia germinating on malt extract agar (MEA) within 24 h. Germ tubes produced from the basal and apical cells of the conidia. Colonies growing on MEA, reaching 40–45 mm in 4 weeks at 25 °C. Mycelia superficial, circular, with entire margin, flat, smooth, from above brown at the center, dark brown at the edge, from below dark brown.

Material examined: THAILAND, Chiang Mai Province, Mushroom Research Center (MRC), on decaying wood submerged in a stream, 13 September 2019, M.S. Calabon, WF03 (MFLU 21–0016, holotype), ex-type living culture, MFLUCC 21–0008.

GenBank accession numbers: LSU = MT860430, SSU = MW485613, ITS = MT864352, *TEF1-α* = MW512606, *RPB2* = MW512609

**Figure 4:**
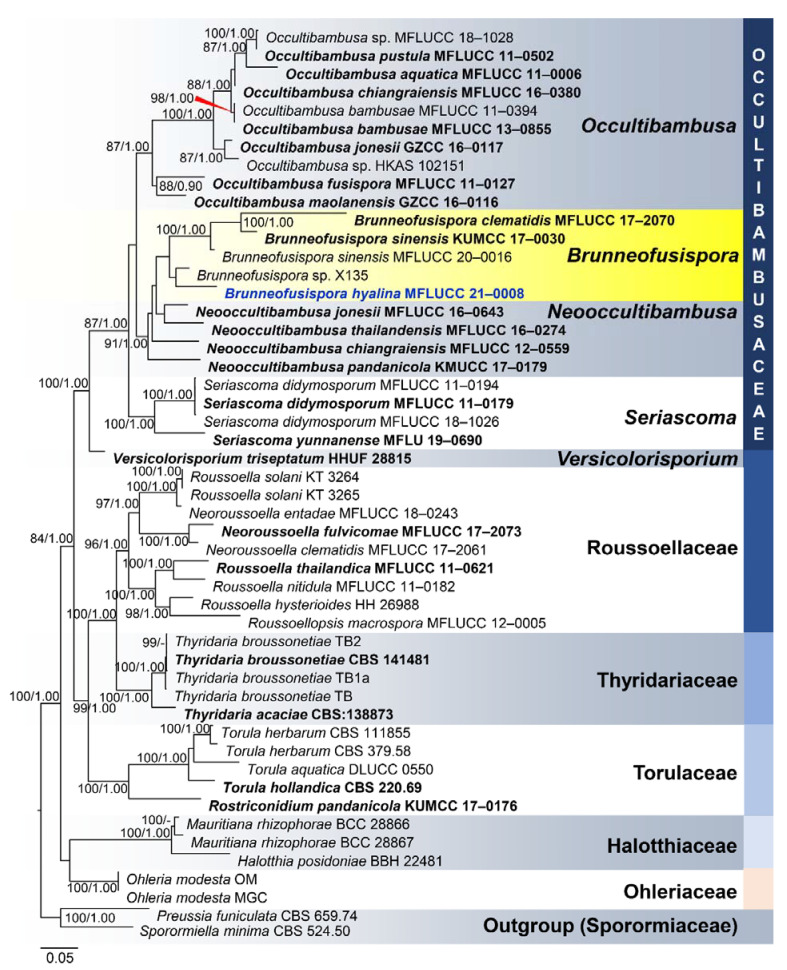
Phylogram generated from maximum likelihood analysis based on combined LSU, SSU, ITS, *TEF1-α*, and *RPB2* sequence data representing the species of Pleosporales. Related sequences are taken from Tibpromma et al. [62]. Fifty taxa were included in the combined analyses, which comprised 6314 characters (LSU = 1116, SSU = 988, ITS = 2438, *TEF1-α* = 678, *RPB2* = 1094) after alignment. The best scoring RAxML tree with a final likelihood value of −22606.054905 is presented. The matrix had 2273 distinct alignment patterns, with 50.65% of undetermined characters or gaps. Estimated base frequencies were as follows: A = 0.249087, C = 0.252571, G = 0.272240, T = 0.226102; substitution rates: AC = 1.279443, AG = 3.002197, AT = 1.311809, CG = 1.122167, CT = 6.076550, GT = 1.000000; gamma distribution shape parameter α = 0.234175. Bootstrap support values for ML equal to or greater than 75% and BYPP equal to or greater than 0.95 are given above the nodes. *Preussia funiculata* CBS 659.74 and *Sporormiella minima* CBS 524.50 in Sporormiaceae were used as the outgroup taxa. The newly generated sequence is indicated in blue. The ex-type strains are indicated in bold.

Notes: Based on morphology, coupled with multi-loci phylogenetic analyses, the novel taxon was placed in *Brunneofusispora*. *Brunneofusispora hyalina* is similar to *B. sinensis* in conidiogenesis and conidial shape but differs in the size of conidiomata (260–435 × 110–205 μm diameter versus 120–160 μm × 80–120 μm diameter) and conidiogenous cells (5–11 μm × 2–4 μm versus 6–7.5 μm × 2.5–3 μm) [60]. In the phylogenetic analysis, *Brunneofusispora hyalina* clustered as a sister taxon to *Brunneofusispora* sp. (X135) (Figure 4). The latter is named in GenBank as *Neooccultibambusa* sp. with an accession number MK304223 for the ITS gene region but there are no available description and photographic plate for this species, so we transferred this to *Brunneofusispora*. BLAST results of ITS and LSU sequence data were Pleosporales sp. E6910l (99% similarity) and *Neooccultibambusa thailandensis* MFLUCC 16–0274 (98% similarity), respectively. The *TEF1-α* sequence was 95% similar to *Brunneofusispora sinensis* (KUMCC 17–0030), *Occultibambusa fusispora* (MFLUCC 11–0127), and *Occultibambusa bambusae* (MFLUCC 13–0855), while *RPB2* sequence data were 85.19% similar to *Occultibambusa fusispora* MFLUCC 11-0127. *Brunneofusispora hyalina* had 35 base pair differences (493 bp, 7.10%) in the ITS region when compared to *Brunneofusispora* sp. (X135). A comparison of ITS and *TEF1-α* sequence data of *B. hyalina* differed by 49 (9.25%, 530 bp) and 37 (6.41%, 577bp) base pairs with *B. clematidis*, respectively. *Brunneofusispora hyalina* differed by 41 (7.87%, 521 bp) and 44 base pairs (4.73%, 931 bp) in ITS and *TEF1-α* sequence data, respectively, when compared to *B. sinensis* (MFLUCC 17–2070).

#### 3.1.2. Sordariomycetes O.E. Erikss. et Winka, Myconet 1: 10 (1997)

The latest treatment of Sordariomycetes by Hyde et al. [16] is followed with 45 orders, 167 families and 1499 genera (with 308 genera *incertae sedis*) listed and described. Three new species of freshwater Sordariomycetes are introduced and described in this paper.

Chaetosphaeriales Huhndorf, A.N. Mill. & F.A. Fernández, Mycologia 96(2): 378 (2004)

Chaetosphaeriales was introduced by Huhndorf et al. [63]. The order comprises five families (Chaetosphaeriaceae, Helminthosphaeriaceae, Leptosporellaceae, Leptosporellaceae, and Linocarpaceae) with 55 genera recorded [49]. Sixty-nine species were recorded from freshwater habitats (Chaetosphaeriaceae: 59, Helminthosphaeriaceae: 6, Linocarpaceae: 4) [10].

Chaetosphaeriaceae Réblová, M.E. Barr & Samuels, Sydowia 51(1): 56 (1999)

Forty-four genera constitute Chaetosphaeriaceae, wherein 12 genera (59 species) were recorded in freshwater habitats [10,49,64]. Freshwater *Dictyochaeta* is the most speciose genus in the family with 16 species recorded [10].

*Rattania* Prabhugaonkar & Bhat, Mycotaxon 108: 218 (2009)

*Rattania* was introduced by Prabhugaonkar and Bhat [65] to accommodates *Rattania setulifera*, a species isolated from fresh leaves of rattan (*Calamus thwaitesii*) in India. Shenoy et al. [66] placed the genus in Chaetosphaeriales. *Rattania* is characterized by sporodochial, setose conidiomata, monoblastic conidiogenous cells and slimy, fusiform, 0–5-septate, setulate conidia [65]. In this paper, a new *Rattania* species observed from submerged decaying wood in freshwater river in Thailand is introduced.

**Figure 5:**
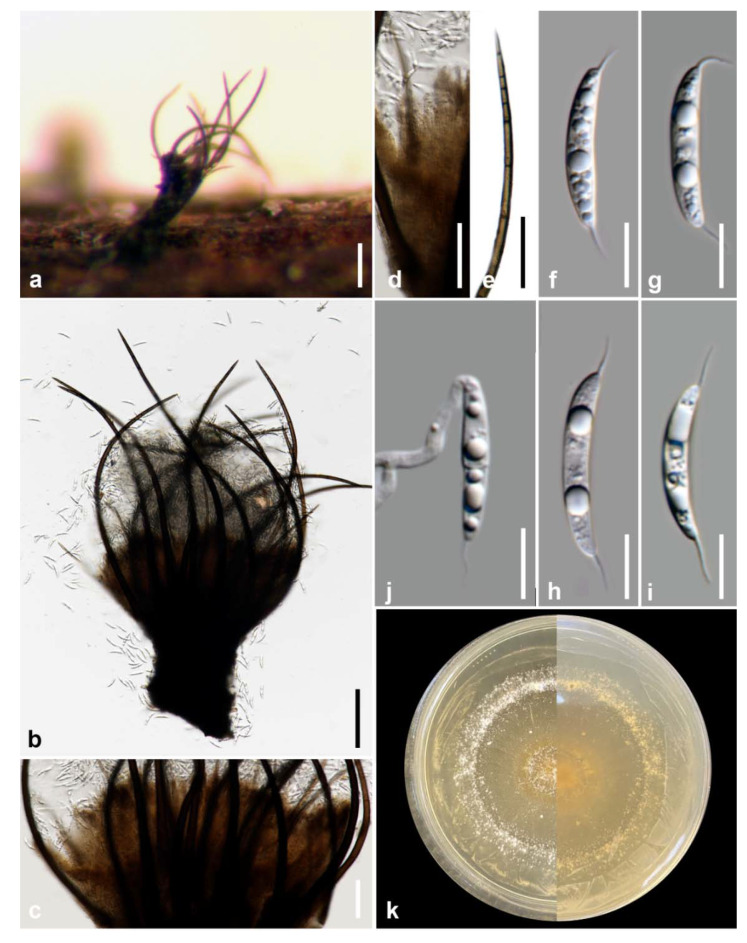
*Rattania aquatica* (MFLU 21–0013, holotype). (**a**) Appearance of conidioma on host; (**b**) synnemata; (**c**,**d**) closely packed conidiophores; (**e**) setae; (**f–i**). conidia; (**j**) germinated conidium; (**k**) culture on MEA. Scale bars: (**a**,**b**) 100 μm; (**c**–**e**) 50 μm; (**f**–**j**) 10 μm.

*Rattania aquatica* M.S. Calabon & K.D. Hyde, sp. nov. (Figure 5)

MycoBank number: MB838553; Facesoffungi number: FoF 09532

Etymology: in reference to the habitat where the fungus was collected

Holotype: MFLU 21–0013

Saprobic on decaying wood submerged in freshwater habitats. **Sexual morph:** Undetermined. **Asexual morph:** *Colonies* on the substrate effuse, scattered, dark brown to black. *Mycelium* mostly immersed, composed of branched, septate, smooth, thin-walled, brown hyphae. *Conidiomata* 170–270 × 55–150 μm ($\bar{x}$ = 210 μm, *n* = 10), superficial, synnematous, scattered, dark brown to black, funnel-shaped, sessile sporodochia with spore mass at the apex. *Setae* 290–475 × 3–14 μm ($\bar{x}$ = 340 × 9, *n* = 10), erect, straight to curved, flexuous, unbranched, irregular in length, cylindrical, tapering towards apex into an acute tip, 10–15 septate, smooth, thick-walled. *Conidiophores* up to 175 μm long, macronematous, brown, cylindrical, densely compacted along the synnematal axis, smooth-walled. *Conidiogenous cells* 2–4 μm long ($\bar{x}$ = 2.8 μm, *n* = 10), terminal, integrated or discrete, hyaline, smooth-walled. *Conidia* 22–27 × 3–5 μm ($\bar{x}$ = 24.9 × 3.9 μm, *n* = 30), hyaline, naviculate to fusiform, apex acute, base truncate, smooth, thin-walled, 0–1 septate, mostly aseptate, with a single filiform setula at both ends, 3.6–8.3 μm long, guttulate.

Culture characteristics: Conidia germinating on malt extract agar (MEA) within 24 h. Germ tubes produced from the basal and apical cell of conidia. Colonies growing on MEA, reaching 30–35 mm in 2 weeks at 25 °C. Mycelia superficial, circular, with entire margin, flat, smooth, from above white, from below white.

Material examined: THAILAND, Tak Province, Tha Sing Yang, Ban Mae Ja Wang on decaying wood submerged in a freshwater river, 17 October 2019, N. Padaruth, CC24 (MFLU 21–0013, holotype), ex-type living culture, MFLUCC 21–0006.

GenBank accession numbers: LSU = MW287235; ITS = MW260331

**Figure 6:**
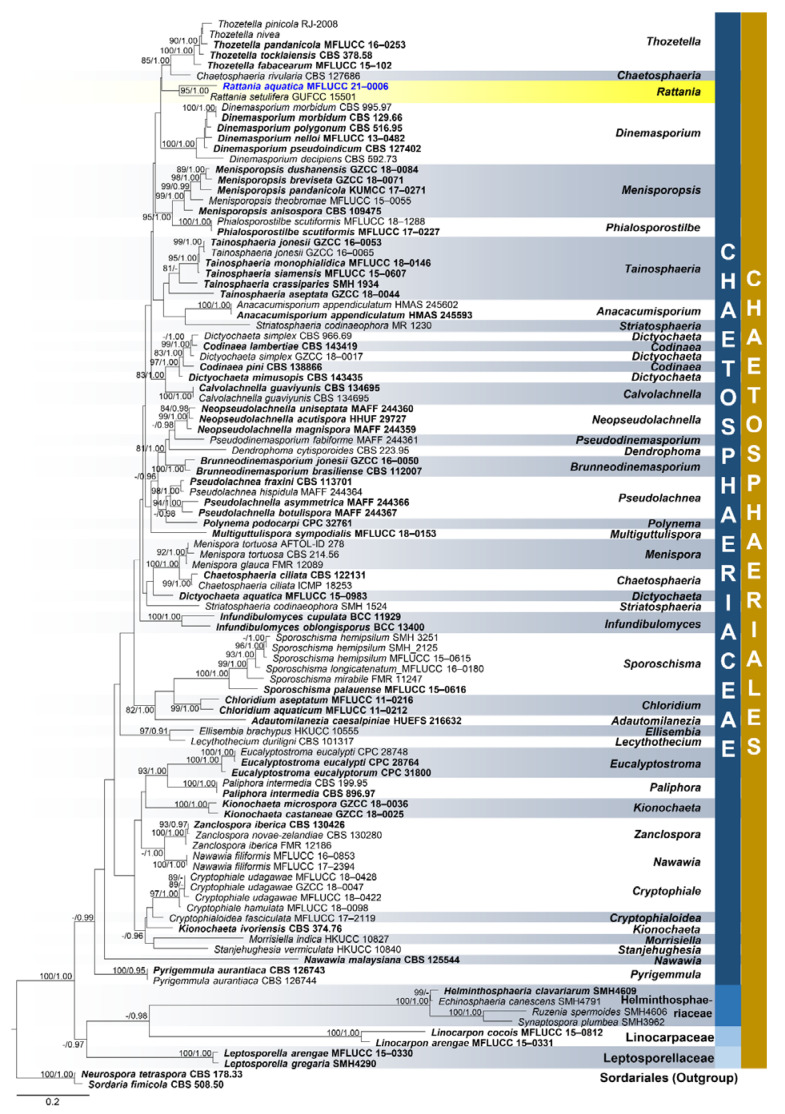
Phylogenetic tree generated from maximum likelihood (ML) analysis based on combined LSU and ITS sequence data for the species from Chaetosphaeriales. Related sequences are taken from Lin et al. [64]. One-hundred three taxa were included in the combined analyses which comprised 1329 characters after alignment including gaps. LSU: 789 bp, ITS: 540 bp. The RAxML analysis of the combined dataset yielded a best scoring tree with a final ML optimization likelihood value of −19,119.372669. The matrix had 730 distinct alignment patterns, with 13.78% undetermined characters or gaps. Estimated base frequencies were as follows: A = 0.225668, C = 0.269306, G = 0.309464, T = 0.195562; substitution rates AC = 1.438984, AG = 1.899345, AT = 1.390083, CG = 0.832127, CT = 6.028381, GT = 1.000000; gamma distribution shape parameter α = 0.286425. Bootstrap support values for ML equal to or greater than 75% and BYPP equal to or greater than 0.95 are given above the nodes. *Neurospora tetraspora* (CBS 178.33) and *Sordaria fimicola* (CBS 508.50) from Sordariales were used as outgroup taxa. The newly generated sequence is indicated in blue. The ex-type strains are indicated in bold.

Notes: *Rattania aquatica* differs from the type species *R. setulifera* in conidiomatal morphology (synnemata versus sporodochia), shorter conidia (22–27 × 3–5 μm versus 5–50 um × 1.5–3.5) and conidial septation (0–1 versus 0–5) [65]. Based on the BLASTn search of ITS sequence data in GenBank, the closest matches are Chaetosphaeriaceae sp. TBA274 (96%) and Sordariomycetes sp. KO-2013 (95%). However, *Rattania setulifera* (GUFCC 15501) is the closest match for the LSU sequence data with 98% similarity. The multi-loci phylogenetic analyses show that *R. aquatica* is a distinct species and sister taxon to *R. setulifera* (GUFCC 15501) with 95% MP, 1.00 BYPP support (Figure 6). A comparison of ITS and LSU sequence data between *R. aquatica* and *R. setulifera* revealed 9.68% (46/475 bp) and 2.20% (18/820 bp) nucleotide base pair differences, respectively.

##### Phyllachorales M.E. Barr, Mycologia 75: 11 (1983)

Phyllachorales was formally described by Barr [67] but phyllachoraceous taxa were placed by several authors in various orders and families (see [68] for historical placement of phyllachoraceous fungi). Four families (Phaeochoraceae, Phaeochorellaceae, Phyllachoraceae, Telimenaceae) and 60 genera were included in Phyllachorales [49]. The divergence time for the order is estimated as 168 MYA (Hyde et al. 2020). Phyllachoraceous taxa are biotrophic, obligate plant parasitic fungi, and saprobic on palms (Arecaceae) and submerged decaying wood [16].

##### Phyllachorales Genera *incertae sedis*

Wijayawardene et al. (2020) listed *Marinosphaera* under Phyllachorales genera *incertae sedis*. In this paper, a novel genus is introduced based on morphology and multi-loci phylogenetic analyses.

*Neoxylomyces* M.S. Calabon, Boonmee, E.B.G. Jones & K.D. Hyde, gen. nov.

MycoBank number: MB838554; Facesoffungi number: FoF 09533

Etymology: referring to the similarity to the genus *Xylomyces*

Saprobic on decaying wood submerged in freshwater habitats. **Sexual morph**: Undetermined. **Asexual morph**: *Colonies* on the substrate effuse, scattered, dark brown to black. *Mycelium* mostly immersed, composed of branched, septate, smooth, thin-walled, dematiaceous, anastomosing hyphae. *Conidiophores* and *conidia* not developed. *Chlamydospores* narrowly fusiform, cylindrical, intercalary, erect, mostly straight, slightly curved, solitary or in chains, occasionally branched, multiseptate, constricted at septa, brown, paler end cells, thick-walled with scarce irregular longitudinal striations.

Type species: *Neoxylomyces multiseptatus* M.S. Calabon, Boonmee, E.B.G. Jones & K.D. Hyde

**Figure 7:**
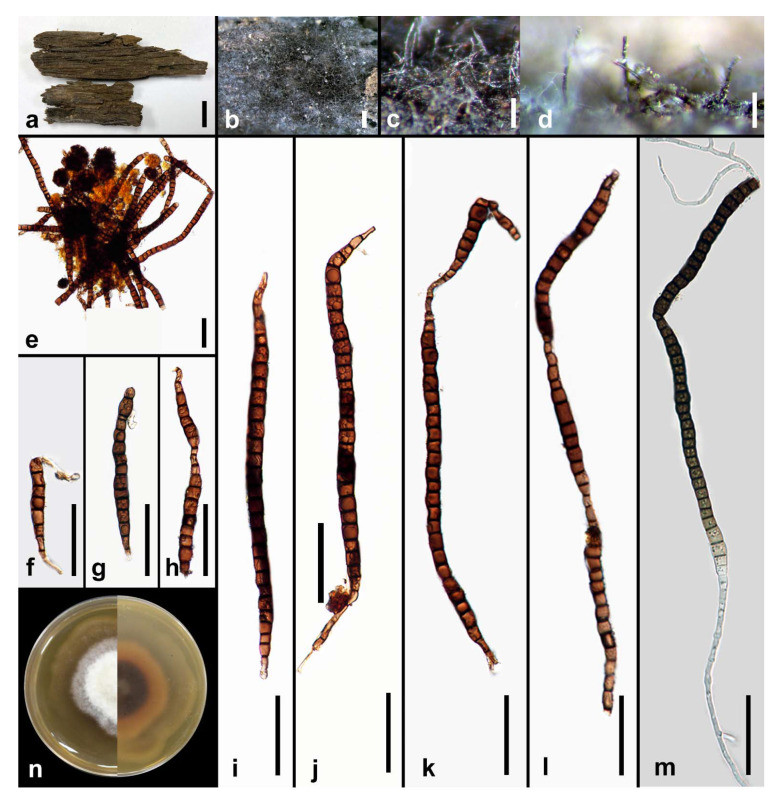
*Neoxylomyces multiseptatus* (MFLU 21–0014, holotype). (**a**) Host; (**b**–**e**) colonies on wood; (**f**–**l**) chlamydospores; (**m**) germinated chlamydospore; (**n**) culture on MEA. Scale bars: (**a**) 10 mm; (**b**,**c**) 500 µm; (**d**) 100 µm; (**e**–**m**) 50 µm.

Notes: *Neoxylomyces* is similar to *Xylomyces* in having brown, thick-walled, multiseptate chlamydospores [14,48]. The latter, typified by *X. chlamydosporus*, is placed in Aliquandostipitaceae while *Neoxylomyces* clustered with other taxa of *Phyllachorales* with 100% ML, 1.00 BYPP support (Figure 7). In multi-loci phylogenetic analyses, *Neoxylomyces* shared the same clade with *Clathrosporium retortum* (CCIBt 4122; CCIBt 4123) with 100% ML, 1.00 BYPP support.

**Figure 8:**
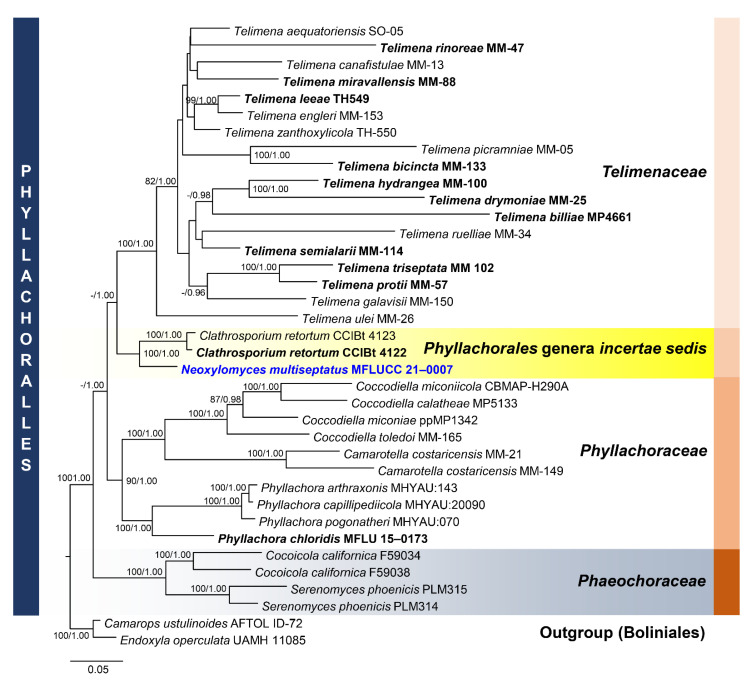
Phylogram generated from maximum likelihood analysis based on combined LSU, SSU, ITS, and *TEF1-α* sequence data representing the species of Phyllachorales and related taxa. Sequences are taken from Dayarathne et al. [72] and Yang et al. [73]. Thirty-seven taxa were included in the combined analyses, which comprised 3259 characters (LSU = 863, SSU = 1175, ITS = 462, *TEF1-α* = 759) after alignment. The best scoring RAxML tree with a final likelihood value of −32,589.920351 is presented. The matrix had 2136 distinct alignment patterns, with 34.35% of undetermined characters or gaps. Estimated base frequencies were as follows: A = 0.248688, C = 0.249512, G = 0.274933, T = 0.226867; substitution rates: AC = 1.044099, AG = 1.599779, AT = 1.069911, CG = 1.048973, CT = 3.108380, GT = 1.000000; gamma distribution shape parameter α = 0.745075. Bootstrap support values for ML equal to or greater than 75% and BYPP equal to or greater than 0.95 are given above the nodes. *Camarops ustulinoides* (AFTOL-ID 72) and *Endoxyla operculata* (UAMH 11085) were used as the outgroup taxa. The newly generated sequence is indicated in blue. The ex-type strains are indicated in bold.

*Neoxylomyces multiseptatus* MS Calabon, Boonmee, E.B.G. Jones & K.D. Hyde, sp. nov. (Figure 8)

MycoBank number: MB838555; Facesoffungi number: FoF 09534

Etymology: In reference to the multiseptate chlamydospores

Holotype: MFLU 21–0014

Saprobic on decaying wood submerged in freshwater habitats. **Sexual morph**: Undetermined. **Asexual morph**: *Colonies* on the substrate effuse, scattered, dark brown to black. *Mycelium* mostly immersed, composed of branched, septate, smooth, thin-walled, dematiaceous, anastomosing hyphae. *Conidiophores* and *conidia* not developed. *Chlamydospores* 111–378 × 8–13 μm ($\bar{x}$ = 255 × 10.2 μm, *n* = 30), narrowly fusiform, cylindrical, intercalary, erect, mostly straight, slightly curved, solitary or in chains, occasionally branched, with 7–45 septa, constricted at septa, brown, paler and truncated end cells, 3.28–5.93 μm wide ($\bar{x}$ = 4.39 μm), thick-walled with scarce irregular longitudinal striations.

Culture characteristics: Chlamydospores germinating on malt extract agar (MEA) within 24 h. Germ tubes produced from the basal and apical cell of conidia. Colonies growing on MEA, reaching 35–40 mm in 4 weeks at 25 °C. Mycelia superficial, circular, with entire margin, flat, smooth, from above ivory to pale brown at the margin, white at the center; reverse, dark brown at the center then becoming pale orange to light brown at the margin.

Material examined: THAILAND, Chiang Mai Province, Mae Teang District, Mushroom Research Center (M.R.C.), on decaying wood submerged in a stream, 11 February 2019, M.S. Calabon, MC02 (MFLU 21–0014, holotype), ex-type living culture, MFLUCC 21–0007.

GenBank accession numbers: LSU = MW287236, SSU = MW287239, ITS = MW260332, *TEF1-α* = MW512607

Notes: *Neoxylomyces multiseptatus* is similar to *Xylomyces giganteus* in having brown, long, multiseptate chlamydospores. The former differs in the number of septa (7–45 versus 6–85), size of the chlamydospores (111–378 × 8–13 μm versus (140) 190–575 × 25–50 μm), and absence of a mucilaginous coating to the chlamydospores [69,70]. Furthermore, *Xylomyces* belongs to Aliquandostipitaceae (Jahnulales, Dothideomycetes) [49]. The closest match of the sequences based on BLASTn searches in NCBI GenBank database were *Clathrosporium*. *Clathrosporium retortum* (CCIBt 4123) was the closest species based on BLAST result of ITS (83% similarity) and LSU sequence data (95% similarity). The *TEF1-α* sequence was 91% similar to *Tolypocladium ophioglossoides* (NBRC:8992), *T. paradoxum* (NBRC:106958)*, Metarhizium granulomatis* (UAMH 11176), and *Hypomyces polyporinus* (ATCC 76479). The multi-loci phylogenetic analyses show that *N. multiseptatus* is a distinct species and sister taxon to *Clathrosporium retortum* (CCIBt 4123) with 100% MP, 1.00 BYPP support. The former has long, narrowly fusiform, multiseptate chlamydospores while the latter has subglobose to irregular, hyaline to subhyaline conidia formed by branched, densely interwoven conidial filaments [71].

Togniniales Senan., Maharachch. & K.D. Hyde, Fungal Diversity 72: 220 (2015)

Maharachchikumbura et al. [74] introduced Togniniales to accommodate Togniniaceae based on multi-loci phylogenetic analyses. The monotypic order is characterized by perithecial ascomata and clavate, tiny asci with hyaline ascogenous hyphae, and cylindrical to allantoid ascospores [74,75]. Hyde et al. [16] estimated the divergence of the order as 138 MYA.

Togniniaceae Réblová, L. Mostert, W. Gams & Crous, Stud. Mycol. 50(2): 540 (2004)

Réblová et al. [76] introduced Togniniaceae based on LSU and SSU sequence data of *Togninia* species. Togniniaceae has been referred to various orders Calosphaeriales [77] and Diaporthales [78], but Maharachchikumbura et al. [74] excluded it from Diaporthales and accommodated Togniniaceae in Togniniales. Two genera, *Conidiotheca* and *Phaeoacremonium*, constitute the family.

*Phaeoacremonium* W. Gams, Crous & M.J. Wingf., Mycologia 88: 789 (1996)

Crous et al. [79] introduced *Phaeoacremonium (=Togninia),* with *P. parasiticum* as the type species. *Phaeoacremonium* was known to be the asexual morph of *Togninia*, a genus introduced by Berlese (1990) with *T. minima* as the type species. *Togninia* was synonymized under *Phaeoacremonium* by Gramaje et al. [80] as the latter has the most species, widely used by mycologist, and some *Togninia* species already have names in *Phaeoacremonium*. Sixty-seven epithets of *Phaeoacremonium* are listed in Species Fungorum (2020; http://www.speciesfungorum.org/Names/Names.asp (accessed on 13 January 2021)).

**Figure 9:**
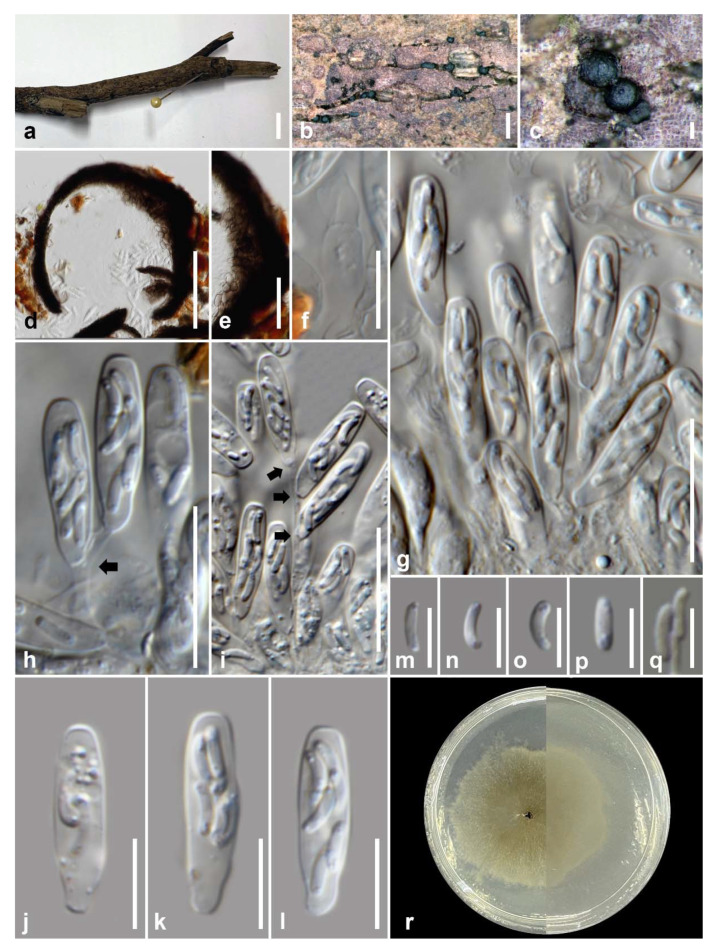
*Phaeoacremonium thailandense* (MFLU 21–0012, holotype). (**a**) Host; (**b**,**c**) ascomata on submerged wood; (**d**) section of an ascoma; (**e**) ascomatal wall; (**f**) paraphyses; (**g**–**i**) ascogenous hyphae (arrows); (**j**–**l**) asci; (**m**–**p**) ascospores; (**q**) germinated ascospores; (**r**) culture on MEA from surface and reverse. Scale bars: (**a**) 10 mm; (**b**) 500 µm; (**c**–**e**) 100 µm; (**f**,**j**–**l**) 10 µm; (**g**–**i**) 20 µm; (**m**–**q**) 5 µm.

*Phaeoacremonium thailandense* M.S. Calabon & K.D. Hyde, sp. nov. (Figure 9)

Mycobank number: MB838556; Facesoffungi number: FoF 09535

Etymology: In reference to the host location, Thailand, where the holotype was collected.

Holotype: MFLU 21–0012

Saprobic on decaying wood submerged in freshwater habitats. **Sexual morph**: *Ascomata* 170–280 µm ($\bar{x}$ = 212 µm, *n* = 10) diameter, perithecial, scattered to gregarious, immersed to semi-immersed, globose to subglobose, black, coriaceous. *Ascomatal wall* 15–45 µm thick ($\bar{x}$ = 28 µm, *n* = 10), membranous, comprising 8–10 layers, of outer dark brown to brown and inner hyaline cells of *textura angularis*. *Hamathecium* composed of 2–7 μm ($\bar{x}$ = 3.9 µm, *n* = 10) wide, hyaline, septate paraphyses, slightly constricted at septa and gradually narrowed towards apex, longer than asci. *Asci* 18–25 × 4–6 µm ($\bar{x}$ = 22 × 5, *n* = 20), 8-spored, unitunicate, clavate, apex truncate, apedicellate, with truncate bases. *Ascogenous hyphae* hyaline, septate, simple, smooth-walled, 2–3 µm at base. *Ascospores* 4.8–6.6 × 1.2–1.6 µm ($\bar{x}$ = 5.7 × 1.4 µm, *n* = 30), biseriate, reniform with rounded ends, unicellular, hyaline, thin-walled, smooth-walled, often containing small guttules at both ends. **Asexual morph**: Undetermined.

Material examined: THAILAND, Tak Province, Tha Sing Yang, Ban Mae Ja Wang, on decaying wood submerged in a freshwater river, 17 October 2019, N. Padaruth, CC12 (MFLU 21–0012, holotype), ex-type living culture, MFLUCC 21–0005.

Culture characteristics: Ascospores germinating on malt extract agar (MEA) within 24 h. Germ tubes produced from the basal and apical cell of conidia. Colonies growing on MEA, reaching 20–25 mm in 2 weeks at 25 °C. Mycelia superficial, circular, with entire margin, flat, smooth, transparent, spare, from above light brown; reverse, light brown.

GenBank numbers: LSU = MW287238, ITS = MW260334, *TEF1-α* = MW512608, *TUB2* = MW512610, *ACT =* MW512611

**Figure 10:**
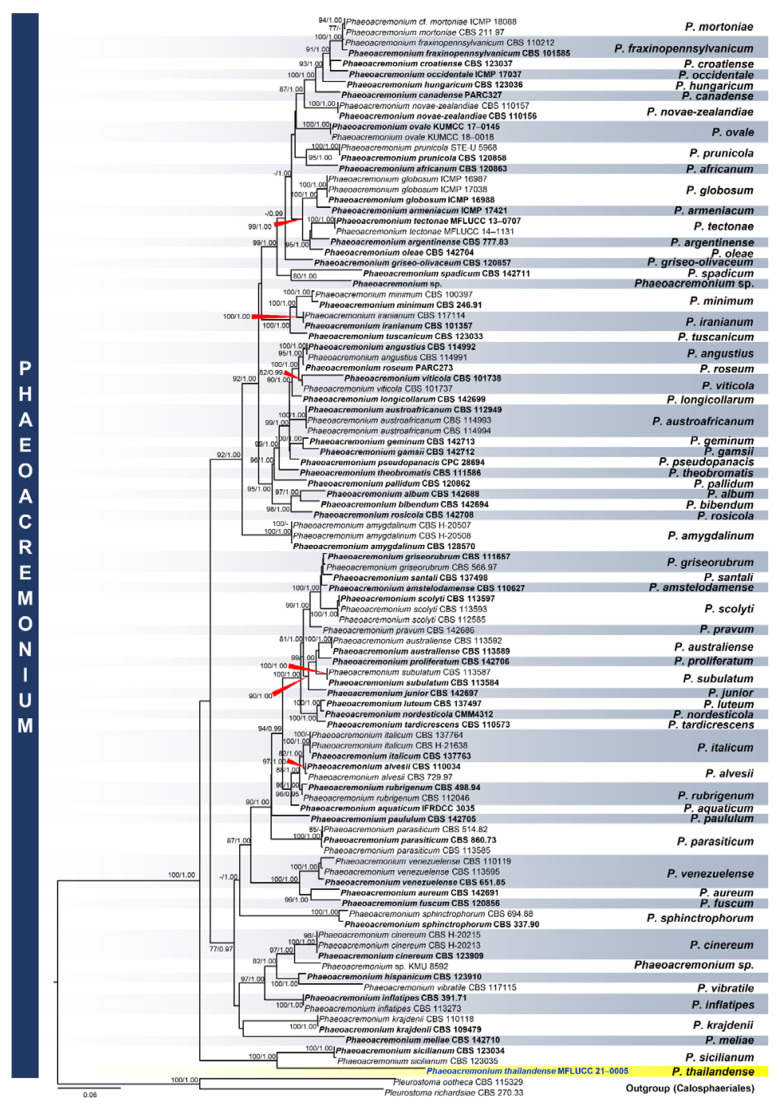
Phylogram generated from maximum likelihood analysis based on combined LSU, SSU, ITS, *TEF1-α*, *TUB2*, and *ACT* sequence data representing the species of Phyllachorales and related taxa. Sequences are taken from Huang et al. [82]. One hundred-two taxa were included in the combined analyses, which comprised 2395 characters (LSU: 892, ITS: 527, *TEF1-α* = 202; *TUB2*: 513; *ACT*: 261) after alignment. The best scoring RAxML tree with a final likelihood value of −22707.720097 is presented. The matrix had 1011 distinct alignment patterns, with 45.51% of undetermined characters or gaps. Estimated base frequencies were as follows: A = 0.228986, C = 0.288393, G = 0.253607, T = 0.229013; substitution rates: AC = 1.333048, AG = 3.557820, AT = 1.416942, CG = 1.385484, CT = 4.971984, GT = 1.000000; gamma distribution shape parameter α = 0.255190. Bootstrap support values for ML equal to or greater than 75% and BYPP equal to or greater than 0.95 are given above the nodes. *Pleurostoma ootheca* (CBS 115329) and *Pleurostoma richardsiae* (CBS 270.33) were used as the outgroup taxa. The newly generated sequence is indicated in blue. The ex-type strains are indicated in bold.

Notes: *Phaeoacremonium thailandense* shares the same morphology with other sexual morphs of the genus, such as ascus formation in acropetal succession, ascal apex thickened without a discharge mechanism, hyaline ascogenous hyphae, and allantoid, reniform to oblong-cylindrical ascospores [16]. Currently, three species of *Phaeoacremonium* are reported in freshwater habitats: *P. aquaticum, P. ovale*, and *P. thailandense* [10]. The novel species differs from *P. aquaticum* and *P. ovale* in the absence of an ostiolar neck, and longer asci (18–25 µm versus 18–21 µm versus 11–20 µm) [81,82]. The closest match of the sequences based on BLASTn searches in GenBank is *Phaeoacremonium*. *Phaeoacremonium sicilianum* (CBS 123034) is the closest species based on BLAST result of ITS region with 92% similarity. The *TEF1-α* sequence was 94% similar to *P. minimum* strains (AFTOL-ID 924; UCRPA7), while *TUB* sequence was 80% similar to *P. silicianum* strains (KER-U-PMS4; KER-U-PMS5; KER-U-PMS6). The multi-loci phylogenetic analyses showed that *P. thailandense* is a distinct species and sister taxon to *P. silicianum* strains (CBS 123034; 123035) with 100% MP, 1.00 BYPP support (Figure 10). A comparison of ITS and *ACT* sequence data between *P. thailandense* and *P. silicianum* revealed 17.45% (41/235 bp) and 6.46% (39/604 bp) nucleotide base pair differences, respectively.

## 4. Discussion

Mycologists in Thailand have initiated a number of projects to document the diversity of freshwater fungi, and this article is a continuation of these studies. Freshwater fungi are an ecological group and include all the major phyla that occur on a wide range of substrates [2]. The first account of Thai freshwater fungi was by Tubaki et al. [83], who listed 40 Ingoldian fungi from foam samples from north and central Thailand. In 1996, BIOTEC initiated a project on lignicolous freshwater fungi under its Fungal Biodiversity Programme, resulting in Sivichai and Boonyene [84] listing 613 freshwater taxa for Thailand. The accumulative annual records of freshwater from 1996–2004 are presented in Figure S1. Zhang et al. [17] listed 173 (in 112 genera) freshwater species (including 34 Ingoldian fungi) published up to the end of 2010. Research on freshwater fungi in China started in the 1920s [85,86,87], and by 2013 [18] listed some 782 species, including 25 chytridiomycetes, 256 ascomycetes, 416 hyphomycetes, 63 oomycetes, and 22 zygomycetes.

We are constantly reminded that an estimated 39.4% of plants are now threatened with extinction, yet 1886 species of fungi were scientifically named for the first time in 2019 (Kew, Report State of the World’s Plants and Fungi). In this article we have focused on the number of new taxa of freshwater fungi introduced over the past five years from samples collected in Thailand. This data shows that there seems no sign of it reaching a plateau [88].

Freshwater ascomycetes have been extensively studied over the past two decades [8,10,14], but it was only in early 2000 that molecular data was used to resolve species [7,9,89,90,91,92]. Monographs and outlines of freshwater Dothideomycetes and Sordariomycetes have been provided by Dong et al. [14] and Luo et al. [10]. One hundred and forty-five genera of freshwater Dothideomycetes (six orders, 43 families) were included, with 32% (46 genera) being unique to freshwater habitats [8,14]. Luo et al. [10] listed 451 species under 160 genera of freshwater Sordariomycetes.

In the present paper, five novel freshwater ascomycetes were introduced based on multi-loci phylogenetic analyses showing the high undiscovered diversity of fungi in lotic habitats in Thailand. Two new freshwater Dothideomycetes, *Camposporium dulciaquae* and *Brunneofusispora hyalina,* add to the increasing number of pleosporalean taxa discovered from freshwater habitats in Thailand. Based on the published works from 2015–2020, 40 novel pleosporolean taxa were discovered: Aigialaceae (1 species) [93], Anteagloniaceae (1 species) [14], Astrosphaeriellaceae (2 species) [14], Dictyosporiaceae (7 species) [14,94,95,96,97], Latoruaceae (2 species) [14,98], Lentitheciaceae (4 species) [20,99,100], Ligninsphaeriaceae (1 species) [15], Lindgomycetaceae (3 species) [14,101], Longipedicellataceae (3 species) [14], Lophiostomataceae (1 species) [14], Melanommataceae (1 species) [97], Morosphaeriaceae (3 species) [14,102], Nigrogranaceae (1 species) [14], Occultibambusaceae (1 species) [103], Parabambusicolaceae (1 species) [14], Phaeosphaeriaceae (1 species) [104], Pleosporales genera *insertae sedis* (2 species) [11,14], Pseudoastrosphaeriellaceae (1 species) [14], Tetraplosphaeriaceae (1 species) [14], Trematosphaeriaceae (1 species) [97], and Wicklowiaceae (2 species) [19,24]. *Camposporium thailandicum* is an addition to the known twelve *Camposporium* species recorded in freshwater habitats: *C. antennatum* [105,106,107], *C. appendiculatum* [27], *C. cambrense* [108,109,110], *C. fusisporum* [111], *C. hyalinum* [112], *C. japonicum* [108,110], *C. marylandicum* [113,114,115], *C. multiseptatum* [27], *C. ontariense* [108], *C. pellucidum* [27,107,108,110,114,115,116], *C. quercicola* [111], and *C. septatum* [27]. *Brunneofusispora hyalina* is the only known species of the genus thriving in freshwater habitats, while other species were collected on different plant hosts like *Clematis subumbellata* in Thailand (*B. clematidis*) [61] and *Magnolia denudata*, and an unknown host in China (*B. sinensis*) [59,60].

Forty new species of freshwater Sordariomycetes were discovered in Thailand over the past five years, and taxa were members of Amphisphaeriales [10], Annulatascales [16], Chaetosphaeriales [10,97], Diaporthomycetidae genera *incertae sedis* [59,117,118], Distoseptisporales [10,16,97,119,120], Magnaporthales [10], Microascales [10,121], Pleurotheciales [10,27,101,118], Pseudodactylariales [27], Savoryellales [27], Sporidesmiales [103], and Xylariales [10]. Three novel Sordariomycetes (*Neoxylomyces multiseptatus, Phaeoacremonium thailandense,* and *Rattania aquatica*) are additions to these species thriving in freshwater habitats in Thailand. *Phaeoacremonium thailandense* is the first reported freshwater species of *Phaeoacremonium* in Thailand. Other freshwater *Phaeoacremonium* species were recorded in China [81,82]. At present, five species, including the novel taxa, of *Phaeoacremonium* were recorded in Thailand and include *P. aureum*, isolated from mangrove plant *Rhizophora mucronata*, *P. parasiticum* causing human diseases [65,122], *P. sphinctrophorum* on dead bamboo culms [58], and *P. tectonae* on *Tectona grandis* [104]. *Neoyxylomyces multiseptatus* is the fourth species under Phyllachorales recorded in freshwater habitats. Other species recorded were *Ascovaginospora stellipala*, *Phyllachora therophila*, *and Tamsiniella labiosa* [8,10]. *Rattania aquatica* is the first member of the genus recorded from freshwater habitats and an addition to 59 species (12 genera) of freshwater Chaetosphaeriaceae.

Between the years 2015–2020, 129 novel species, dominated by Dothideomycetes with 86 species, followed by Sordariomycetes (40 species), have been discovered from freshwater habitats in Thailand (Figure S2, Table S1). Most of freshwater fungi were collected from streams, which reflects the ease of sampling in these sites. All these freshwater taxa were saprobes in submerged decaying woods. Most have an asexual morph form, wherein 80 and 4 species were hyphomycetes and coelomycetes, respectively. Thirty-five species were sexual morphs, and 10 species had both sexual-asexual morphs. The discovery of five novel freshwater ascomycetes (1 sexual morph, 4 asexual morphs) add to the increasing number of fungi discovered in Thailand for the past six years (Figure S3). Future work needs to explore various lentic and lotic habitats and different substrates in freshwater environments of Thailand. While the Ascomycota dominate taxa recovered from freshwater habitats, much remains to be done to survey other major taxa and develop better techniques for their enumeration, such as members of the Basidiomycota (only 115 are listed by Jones et al. [2]), Zygomycota, Mucoromycota, and microsporidians [2]. These groups must be covered so as to determine the overall diversity of freshwater fungi in Thailand.

## Data Availability

All sequences generated in this study were submitted to GenBank.

## Supplementary Materials

The following are available online at https://www.mdpi.com/2309-608X/7/2/117/s1, Table S1: Freshwater fungi discovered from 2015–2020 in Thailand, Figure S1: Annual cumulative number of freshwater fungi recorded for Thailand from 1996–2004. (Adapted from Sivichai and Boonyene), Figure S2: Classification of novel freshwater fungi discovered from Thailand from 2015–2020, Figure S3: Total number of novel freshwater fungi discovered from Thailand from 2015–2020.
