# Supplementary material for: Five Novel Freshwater Ascomycetes Indicate High Undiscovered Diversity in Lotic Habitats in Thailand
Source: J Fungi (Basel). 2021 Feb 5;7(2):117. doi: 10.3390/jof7020117 (PMC7914987; doi:10.3390/jof7020117)
Supplement: Supplementary file 1 [file jof-07-00117-s001.pdf]

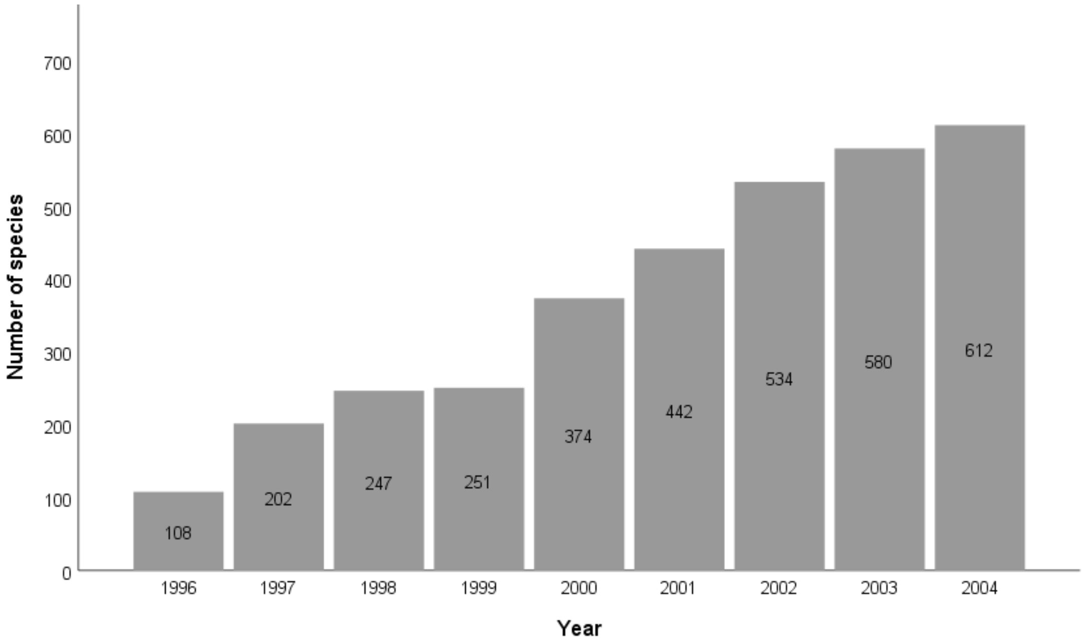

Figure S1. Annual cumulative number of freshwater fungi recorded for Thailand from 1996-2004. (Adapted from Sivichai and Boonyene [1])

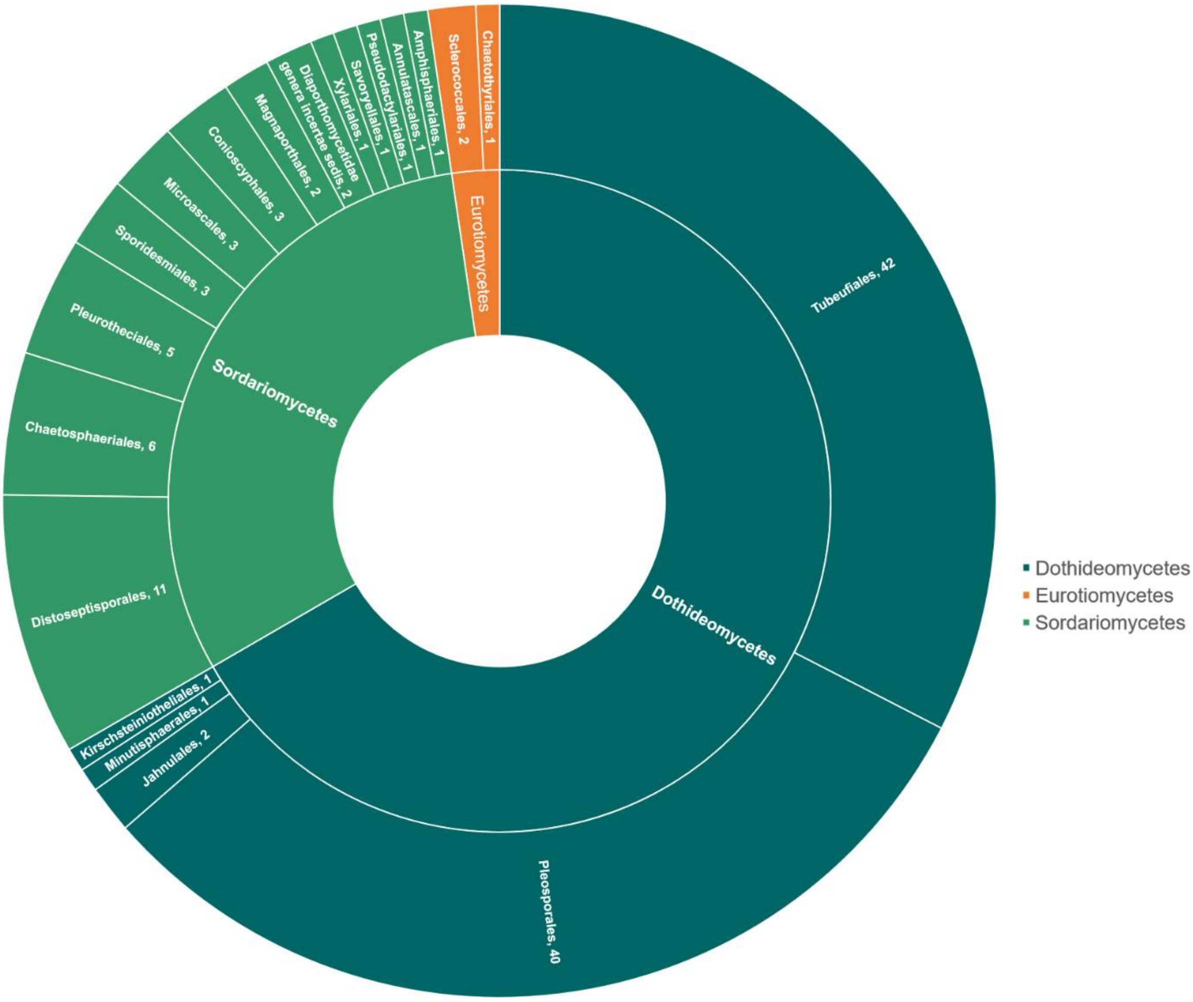

Figure S2. Classification of novel freshwater fungi discovered from Thailand from 2015–2020.

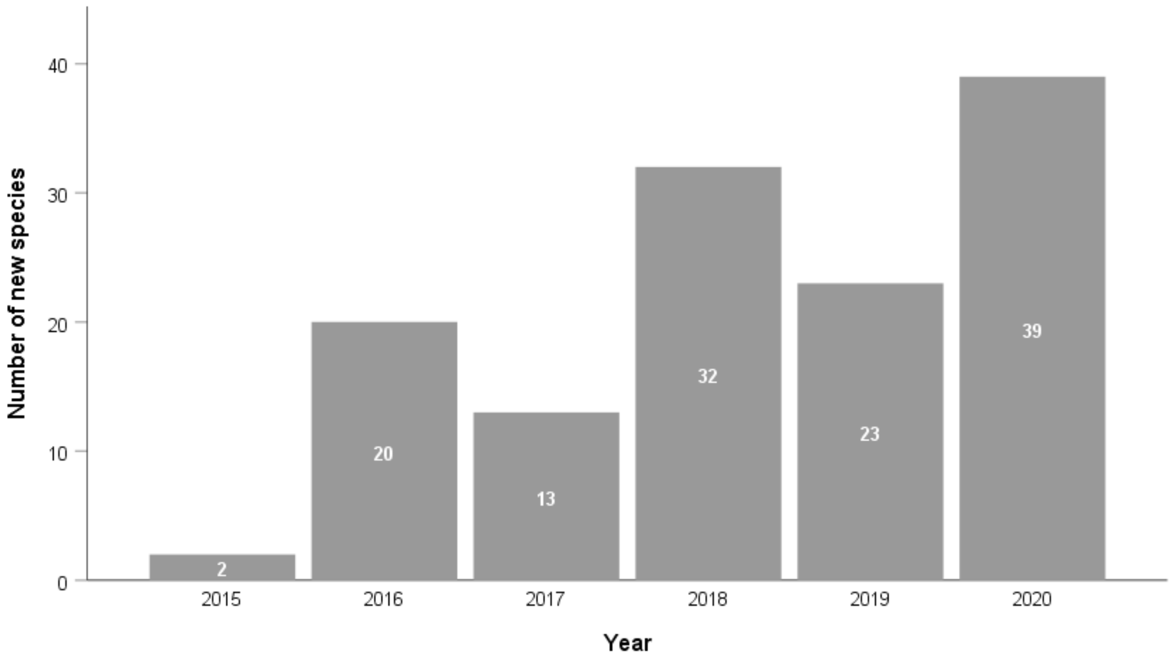

Figure S3. Total number of novel freshwater fungi discovered from Thailand from 2015–2020.

Table S1. Freshwater fungi discovered from 2015–2020 in Thailand.

Species	Habitat	Year	Life mode	Morph	References
**DOTHIDEOMYCETES**					
**JAHNULALES**					
**Aliquandostipitaceae**					
Ascagilis submersa	Stream	2020	Saprobic	Sexual	[2]
Ascagilis thailandensis	Stream	2020	Saprobic	Sexual	[2]
**KIRSCHSTEINIOTHELIALES**					
**Kirschsteiniotheliaceae**					
Kirschsteiniothelia rostrata	Stream	2017	Saprobic	Asexual^{+}	[3]
**MINUTISPHAERALES**					
**Acrogenosporaceae**					
Acrogenospora thailandica	Stream	2019	Saprobic	Asexual^{+}	[4]
**PLEOSPORALES**					
**Aigialaceae**					
Neoastrosphaeriella aquatica	-	2019	Saprobic	Sexual	[5]
**Anteagloniaceae**					
Purpureofaciens aquatica	Stream	2020	Saprobic	Sexual	[2]
**Astrosphaeriellaceae**					
Aquatospora cylindrica	Stream	2020	Saprobic	Sexual	[2]
Caryospora submersa	Stream	2020	Saprobic	Sexual	[2]
**Dictyosporiaceae**					
Dictyocheirospora rotunda	Stream	2016	Saprobic	Asexual^{+}	[6]
Dictyocheirospora thailandica	Stream	2020	Saprobic	Asexual^{+}	[2]
Dictyocheirospora vinaya	Stream	2016	Saprobic	Asexual^{+}	[6]
Dictyosporium thailandicum	Stream	2015	Saprobic	Asexual^{+}	[7]
Dictyosporium tratense	Stream	2018	Saprobic	Asexual^{+}	[82]
Dictyosporium tubulatum	Stream	2018	Saprobic	Asexual^{+}	[82]
Digitodesmium chiangmaiense	Stream	2019	Saprobic	Asexual^{+}	[4]
**Latoruaceae**					

Species	Habitat	Year	Life mode	Morph	References
Pseudoasteromassaria aquatica	Stream	2020	Saprobic	Asexual*	[2]
Pseudoasteromassaria spadicea	Stream	2017	Saprobic	Asexual*	[8]
**Lentitheciaceae**					
Halobyssothecium bambusicola	Stream	2020	Saprobic	Asexual*	[20]
Poaceascoma aquaticum	Stream	2016	Saprobic	Sexual	[9]
Tingoldiago clavata	-	2020	Saprobic	Sexual	[10]
Tingoldiago hydei	-	2020	Saprobic	Sexual	[10]
**Ligninsphaeriaceae**					
Ligninsphaeriopsis thailandica	Stream	2020	Saprobic	Sexual	[11]
**Lindgomycetaceae**					
Hongkongmyces aquaticus	Stream	2020	Saprobic	Asexual*	[2]
Hongkongmyces thailandica	River	2017	Saprobic	Sexual	[3]
Lindgomyces aquaticus	Stream	2020	Saprobic	Sexual	[2]
**Longipedicellataceae**					
Longipedicellata aquatica	Stream	2020	Saprobic	Sexual	[2]
Pseudoxylomyces aquaticus	Stream	2020	Saprobic	Asexual+	[2]
Submersispora variabilis	Stream	2020	Saprobic	Asexual+	[2]
**Lophiostomataceae**					
Lentistoma aquaticum	Stream	2020	Saprobic	Sexual	[2]
**Melanommataceae**					
Camposporium septatum	Stream	2020	Saprobic	Asexual+	[12]
**Morosphaeriaceae**					
Aquihelicascus songkhlaensis	Stream	2020	Saprobic	Sexual	[2]
Helicascus chiangraiensis	Pond	2016	Saprobic	Sexual	[13]
Helicascus uniseptatus	Stream	2016	Saprobic	Sexual	[13]
**Nigrogranaceae**					
Nigrograna aquatica	Stream	2020	Saprobic	Asexual+	[2]
**Occultibambusaceae**					
Occultibambusa aquatica	Waterfall	2016	Saprobic	Sexual	[14]
**Parabambusicolaceae**					
Parabambusicola aquatica	Stream	2020	Saprobic	Sexual	[2]
**Phaeosphaeriaceae**					
Ophiosphaerella aquaticus	Stream	2015	Saprobic	Sexual	[15]
**Pleosporales genera *insertae sedis***					
Mycoenterolobium aquadictyosporium	Stream	2020	Saprobic	Asexual+	[16]
Fusiformiseptata crocea	Stream	2020	Saprobic	Sexual	[2]
**Pseudoastrosphaeriellaceae**					
Pseudoastrosphaeriella aquatica	Stream	2020	Saprobic	Sexual	[2]
**Tetraplosphaeriaceae**					
Shrungabeeja aquatica	Stream	2020	Saprobic	Asexual+	[2]
**Trematosphaeriaceae**					
Falciformispora aquatica	Waterfall	2019	Saprobic	Sexual	[4]
**Wicklowiaceae**					
Wicklowia phuketensis	Stream	2020	Saprobic	Sexual	[19]
Wicklowia submersa	Stream	2019	Saprobic	Sexual	[24]
**TUBEUFIALES**					
**Tubeufiaceae**					
Berkleasmium longisporum	Stream	2018	Saprobic	Asexual+	[17]
Chlamydotubeufia aquatica		2017	Saprobic	Sexual/Asexual+	[18]
Chlamydotubeufia cylindrica	Stream	2018	Saprobic	Sexual/Asexual+	[17]

Species	Habitat	Year	Life mode	Morph	References
Chlamydotubeufia krabiensis	Stream	2017	Saprobic	Sexual/Asexual⁺	[3]
Dictyospora thailandica		2017	Saprobic	Sexual/Asexual⁺	[18]
Helicoarctatus aquaticus	Stream	2018	Saprobic	Asexual⁺	[17]
Helicoarctatus thailandicus	Stream	2020	Saprobic	Asexual⁺	[19]
Helicodochium aquaticum	Stream	2018	Saprobic	Asexual⁺	[17]
Helicohyalinum infundibulum	Stream	2018	Saprobic	Asexual⁺	[17]
Helicoma brunneisporum	Stream	2018	Saprobic	Asexual⁺	[17]
Helicoma longisporum	Stream	2018	Saprobic	Asexual⁺	[17]
Helicoma septoconstrictum	Stream	2018	Saprobic	Asexual⁺	[17]
Helicosporium aquaticum	Stream	2018	Saprobic	Asexual⁺	[17]
Helicosporium flavisporum	Stream	2018	Saprobic	Asexual⁺	[17]
Helicosporium flavum		2017	Saprobic	Sexual/Asexual⁺	[18]
Helicosporium setiferum	Stream	2018	Saprobic	Asexual⁺	[17]
Helicosporium thailandense	Stream	2020	Saprobic	Asexual⁺	[2]
Helicosporium vesicarium	Stream	2018	Saprobic	Asexual⁺	[17]
Helicotubeufia hydei	Stream	2019	Saprobic	Sexual	[20]
Neoacanthostigma brunneisporum	Stream	2017	Saprobic	Sexual/Asexual⁺	[21]
Neoacanthostigma latisporum	Stream	2017	Saprobic	Asexual⁺	[21]
Neochlamydotubeufia fusiformis	Stream	2018	Saprobic	Sexual/Asexual⁺	[17]
Neohelicomyces hyalosporus	Stream	2018	Saprobic	Sexual/Asexual⁺	[17]
Neohelicomyces thailandicus	Stream	2020	Saprobic	Asexual⁺	[2]
Neohelicosporium acrogenisporum	Stream	2018	Saprobic	Asexual⁺	[17]
Neohelicosporium astrictum	Stream	2018	Saprobic	Asexual⁺	[17]
Neohelicosporium ellipsoideum	Stream	2018	Saprobic	Sexual/Asexual⁺	[17]
Neohelicosporium krabiense	Stream	2018	Saprobic	Asexual⁺	[17]
Neohelicosporium submersum	Stream	2020	Saprobic	Asexual⁺	[2]
Neohelicosporium thailandicum	Stream	2017	Saprobic	Asexual⁺	[22]
Pseudohelicomyces aquaticus	Stream	2018	Saprobic	Asexual⁺	[17]
Tubeufia abundata	Stream	2018	Saprobic	Asexual⁺	[17]
Tubeufia brevis	Stream	2018	Saprobic	Asexual⁺	[17]
Tubeufia brunnea	Stream	2018	Saprobic	Asexual⁺	[17]
Tubeufia chlamydospora	Stream	2018	Saprobic	Asexual⁺	[17]
Tubeufia filiformis	Stream	2016	Saprobic	Sexual/Asexual⁺	[23]
Tubeufia inaequalis	Stream	2018	Saprobic	Asexual⁺	[17]
Tubeufia krabiensis	Stream	2018	Saprobic	Asexual⁺	[17]
Tubeufia latispora	Stream	2016	Saprobic	Sexual	[23]
Tubeufia laxispora	Stream	2016	Saprobic	Asexual⁺	[23]
Tubeufia mackenziei	Stream	2016	Saprobic	Asexual⁺	[23]
Tubeufia tratensis	Stream	2018	Saprobic	Asexual⁺	[17]
**EUROTIOMYCETES**					
**SCLEROCOCCALES**					
**Dactylosporaceae**					
Cylindroconidiis aquaticus	Stream	2018	Saprobic	Asexual⁺	[24]

Species	Habitat	Year	Life mode	Morph	References
Pseudobactrodesmium chiangmaiensis	Stream	2020	Saprobic	Asexual+	[26]
**CHAETOTHYRIALES**					
**Herpotrichiellaceae**					
Thysanorea uniseptata	Stream	2019	Saprobic	Asexual+	[4]
**SORDARIOMYCETES**					
**AMPHISPHAERIALES**					
**Sporocadaceae**					
Lepteutypa aquatica	Stream	2019	Saprobic	Sexual	[25]
**ANNULATASCALES**					
**Annulatascaceae**					
Annulatascus thailandensis	Stream	2020	Saprobic	Sexual	[26]
**CHAETOSPHAERIALES**					
**Chaetosphaeriaceae**					
Dictyochaeta siamensis	Stream	2016	Saprobic	Asexual+	[27]
Sporoschisma chiangraiense	Stream	2019	Saprobic	Asexual+	[4]
Sporoschisma palauense	Stream	2016	Saprobic	Asexual+	[28]
Sporoschisma longicatenatum	Stream	2016	Saprobic	Asexual+	[28]
Tainosphaeria obclavata	Stream	2019	Saprobic	Asexual+	[25]
Tainosphaeria siamensis	Stream	2016	Saprobic	Asexual+	[27]
**CONIOSCYPHALES**					
**Conioscyphaceae**					
Conioscypha nakagirii	Stream	2016	Saprobic	Asexual+	[29]
Tretoheliocephala cylindrospora	Stream	2019	Saprobic	Asexual+	[30]
**Barbatosphaeriaceae**					
Barbatosphaeria aquatica	Stream	2018	Saprobic	Asexual+	[31]
**Diaporthomycetidae genera *incertae sedis***					
Aquimonospora tratensis	Stream	2019	Saprobic	Asexual+	[32]
Proliferophorum thailandicum	Stream	2019	Saprobic	Asexual+	[33]
**DISTOSEPTISPORALES**					
**Distoseptisporaceae**					
Distoseptispora appendiculata	Stream	2019	Saprobic	Asexual+	[25]
Distoseptispora lignicola	Waterfall	2019	Saprobic	Asexual+	[25]
Distoseptispora multiseptata	Stream	2016	Saprobic	Asexual+	[14]
Distoseptispora neorostrata	Stream	2019	Saprobic	Asexual+	[25]
Distoseptispora obclavata	Stream	2019	Saprobic	Asexual+	[25]
Distoseptispora rayongensis	Stream	2020	Saprobic	Asexual+	[26]
**Fuscosporellaceae**					
Fuscosporella aquatica	Stream	2017	Saprobic	Asexual+	[34]
Mucispora infundibulata	Stream	2020	Saprobic	Asexual+	[26]
Mucispora phangngaensis	Stream	2017	Saprobic	Asexual+	[34]
Parafuscosporella aquatica	River	2020	Saprobic	Asexual+	[35]
Parafuscosporella pyriformis	River	2020	Saprobic	Asexual+	[35]
**MAGNAPORTHALES**					
**Ceratosphaeriaceae**					
Ceratosphaeria lignicola	Stream	2019	Saprobic	Sexual	[25]
**Ophioceraceae**					
Ophioceras submersum	Waterfall	2019	Saprobic	Sexual	[10]
**MICROASCALES**					
**Halosphaeriaceae**					
Aniptodera aquibella	Stream	2016	Saprobic	Sexual	[36]

Species	Habitat	Year	Life mode	Morph	References
Ascosacculus fusiformis	Stream	2019	Saprobic	Sexual	[25]
**Triadelphialaceae**					
Triadelphia hexaformispora	Stream	2019	Saprobic	Asexual+	[37]
**PLEUROTHECIALES**					
**Pleurotheciaceae**					
Neomonodictys muriformis	Stream	2020	Saprobic	Asexual+	[12]
Phaeoisaria filiformis	Waterfall	2019	Saprobic	Sexual	[25]
Pleurotheciella krabiensis	Stream	2018	Saprobic	Asexual+	[31]
Pleurotheciella tropica	Stream	2018	Saprobic	Asexual+	[31]
Pleurothecium floriforme	Stream	2017	Saprobic	Asexual+	[3]
**PSEUDODACTYLARIALES**					
**Pseudodactylariaceae**					
Pseudodactylaria camporesiana	Stream	2020	Saprobic	Asexual+	[12]
**SAVORYELLALES**					
**Savoryellaceae**					
Canalisporium aquaticium	Stream	2020	Saprobic	Asexual+	[12]
**SPORIDESMIALES**					
**Sporidesmiaceae**					
Sporidesmium aquaticivaginatum	Stream	2016	Saprobic	Asexual+	[14]
Sporidesmium olivaceoconidium	Stream	2016	Saprobic	Asexual+	[14]
Sporidesmium pyriformatum	Stream	2016	Saprobic	Asexual+	[14]
**XYLARIALES**					
**Diatrypaceae**					
Peroneutypa lignicola	Stream	2019	Saprobic	Sexual	[25]

+ Hyphomycete

* Coelomycete

References

1. Sivichai, S.; Boonyene, N. Freshwater fungi. In *Thai Fungal Diversity*; Jones, E.B.G., Tanticharoen, M., Hyde, K.D., Eds.; BIOTEC: Thailand, 2004; pp. 95–106.
2. Dong, W.; Wang, B.; Hyde, K.D.; McKenzie, E.H.C.; Raja, H.A.; Tanaka, K.; Abdel-Wahab, M.A.; Abdel-Aziz, F.A.; Doilom, M.; Phookamsak, R.; et al. Freshwater Dothideomycetes. *Fungal Divers.* **2020**.
3. Hyde, K.D.; Norphanphoun, C.; Abreu, V.P.; Bazzicalupo, A.; Thilini Chethana, K.W.; Clericuzio, M.; Dayarathne, M.C.; Dissanayake, A.J.; Ekanayaka, A.H.; He, M.Q.; et al. Fungal diversity notes 603–708: taxonomic and phylogenetic notes on genera and species. *Fungal Divers.* **2017**, *87*, 1–235, doi:10.1007/s13225-017-0391-3.
4. Hyde, K.D.; Tennakoon, D.S.; Jeewon, R.; Bhat, D.J.; Maharachchikumbura, S.S.N.; Rossi, W.; Leonardi, M.; Lee, H.B.; Mun, H.Y.; Houbraken, J.; et al. Fungal diversity notes 1036–1150: taxonomic and phylogenetic contributions on genera and species of fungal taxa. *Fungal Divers.* **2019**, *96*, 1–242, doi:10.1007/s13225-019-00429-2.
5. Bao, D.F.; Luo, Z.L.; Jeewon, R.; Nalumpang, S.; Su, H.Y.; Hyde, K.D. *Neoastrosphaeriella aquatica* sp. nov. (Aigialaceae), a new species from freshwater habitat in Southern Thailand. *Phytotaxa* **2019**, *391*, 197–206, doi:10.11646/phytotaxa.391.3.3.
6. Boonmee, S.; D'souza, M.J.; Luo, Z.; Pinruan, U.; Tanaka, K.; Su, H.; Bhat, D.J.; McKenzie, E.H.C.; Jones, E.B.G.; Taylor, J.E.; et al. Dictyosporiaceae fam. nov. *Fungal Divers.* **2016**, *80*, 457–482, doi:10.1007/s13225-016-0363-z.
7. Liu, J.K.; Hyde, K.D.; Jones, E.B.G.; Ariyawansa, H.A.; Bhat, D.J.; Boonmee, S.; Maharachchikumbura, S.S.N.; McKenzie, E.H.C.; Phookamsak, R.; Phukhamsakda, C.; et al. Fungal diversity notes 1–110: taxonomic and phylogenetic contributions to fungal species. *Fungal Divers.* **2015**, *72*, 1–197, doi:10.1007/s13225-015-0324-y.
8. Tibpromma, S.; Hyde, K.D.; Jeewon, R.; Maharachchikumbura, S.S.N.; Liu, J.K.; Bhat, D.J.; Jones, E.B.G.; McKenzie, E.H.C.; Camporesi, E.; Bulgakov, T.S.; et al. Fungal diversity notes 491–602: taxonomic and

phylogenetic contributions to fungal taxa. *Fungal Divers.* **2017**, *83*, 1–261, doi:10.1007/s13225-017-0378-0.
9. Luo, Z.L.; Bahkali, A.H.; Liu, X.Y.; Phookamsak, R.; Zhao, Y.C.; Zhou, D.Q.; Su, H.Y.; Hyde, K.D. *Poaceascoma aquaticum* sp. nov. (Lentitheciaceae), a new species from submerged bamboo in freshwater. *Phytotaxa* **2016**, *253*, 71–80, doi:10.11646/phytotaxa.253.1.5.
10. Xu, L.; Bao, D.F.; Luo, Z.L.; Su, X.J.; Shen, H.W.; Su, H.Y. Lignicolous freshwater ascomycota from Thailand: Phylogenetic and morphological characterisation of two new freshwater fungi: *Tingoldiago hydei* sp. nov. and *T. clavata* sp. nov. and eastern Thailand. *MycoKeys* **2020**, *65*, 119–138, doi:10.3897/mycokeys.65.49769.
11. Hongsanan, S.; Hyde, K.D.; Phookamsak, R.; Wanasinghe, D.N.; McKenzie, E.H.C.; Sarma, V. V.; Boonmee, S.; Lücking, R.; Bhat, D.J.; Liu, N.G.; et al. Refined families of Dothideomycetes: Dothideomycetidae and Pleosporomycetidae. *Mycosphere* **2020**, *11*, 1553–2107, doi:10.5943/MYCOSPHERE/11/1/13.
12. Hyde, K.D.; Dong, Y.; Phookamsak, R.; Jeewon, R.; Bhat, D.J.; Jones, E.B.G.; Liu, N.G.; Abeywickrama, P.D.; Mapook, A.; Wei, D.; et al. Fungal diversity notes 1151–1276: taxonomic and phylogenetic contributions on genera and species of fungal taxa. *Fungal Divers.* **2020**, *100*, 5–277, doi:10.1007/s13225-020-00439-5.
13. Luo, Z.L.; Yang, J.; Liu, J.K.; Su, H.Y.; Bahkali, A.H.; Hyde, K.D. Two new species of *Helicascus* (Morosphaeriaceae) from submerged wood in northern Thailand. *Phytotaxa* **2016**, *270*, 182–190, doi:10.11646/phytotaxa.270.3.2.
14. Hyde, K.D.; Hongsanan, S.; Jeewon, R.; Bhat, D.J.; McKenzie, E.H.C.; Jones, E.B.G.; Phookamsak, R.; Ariyawansa, H.A.; Boonmee, S.; Zhao, Q.; et al. Fungal diversity notes 367–490: taxonomic and phylogenetic contributions to fungal taxa. *Fungal Divers.* **2016**, *80*, 1–270, doi:10.1007/s13225-016-0373-x.
15. Ariyawansa, H.A.; Hyde, K.D.; Jayasiri, S.C.; Buyck, B.; Chethana, K.W.T.; Dai, D.Q.; Dai, Y.C.; Daranagama, D.A.; Jayawardena, R.S.; Lücking, R.; et al. Fungal diversity notes 111–252—taxonomic and phylogenetic contributions to fungal taxa. *Fungal Divers.* **2015**, *75*, 27–274, doi:10.1007/s13225-015-0346-5.
16. Calabon, M.S.; Hyde, K.D.; Jones, E.B.G.; Doilom, M.; Liao, C.F.; Boonmee, S. *Mycoenterolobium aquadictyosporium* sp. nov. (Pleosporomycetidae, Dothideomycetes) from a freshwater habitat in Thailand. *Mycol. Prog.* **2020**, *19*, 1031–1042, doi:10.1007/s11557-020-01609-0.
17. Lu, Y.Z.; Liu, J.K. (Jack); Hyde, K.D.; Jeewon, R.; Kang, J.C.; Fan, C.; Boonmee, S.; Bhat, D.J.; Luo, Z.L.; Lin, C.G.; et al. A taxonomic reassessment of Tubeufiales based on multi-locus phylogeny and morphology. *Fungal Divers.* **2018**, *92*, 131–344, doi:10.1007/s13225-018-0411-y.
18. Brahmanage, R.S.; Lu, Y.Z.; Bhat, D.J.; Wanasinghe, D.N.; Yan, J.Y.; Hyde, K.D.; Boonmee, S. Phylogenetic investigations on freshwater fungi in Tubeufiaceae (Tubeufiales) reveals the new genus *Dictyospora* and new species *Chlamydotubeufia aquatica* and *Helicosporium flavum*. *Mycosphere* **2017**, *8*, 917–933, doi:10.5943/MYCOSPHERE/8/7/8.
19. Yuan, H.S.; Lu, X.; Dai, Y.C.; Hyde, K.D.; Kan, Y.H.; Kušan, I.; He, S.H.; Liu, N.G.; Sarma, V.V.; Zhao, C.L.; et al. Fungal diversity notes 1277–1386: taxonomic and phylogenetic contributions to fungal taxa. *Fungal Divers.* **2020**, *104*, 1277–1386, doi:10.1007/s13225-020-00461-7.
20. Liu, J.K.; Lu, Y.Z.; Cheewangkoon, R.; To-Anun, C. Phylogeny and morphology of *Helicotubeufia* gen. nov., with three new species in Tubeufiaceae from aquatic habitats. *Mycosphere* **2018**, *9*, 495–509, doi:10.5943/mycosphere/9/3/4.
21. Lu, Y.Z.; Boonmee, S.; Liu, J.K.; Hyde, K.D.; Bhat, D.J.; Eungwanichayapant, P.D.; Kang, J.C. Novel *Neoacanthostigma* species from aquatic habitats. *Cryptogam. Mycol.* **2017**, *38*, 169–190, doi:10.7872/crym/v38.iss2.2017.169.
22. Lu, Y.Z.; Boonmee, S.; Liu, J.K.; Hyde, K.D.; McKenzie, E.H.C.; Eungwanichayapant, P.D.; Kang, J.C. Multi-gene phylogenetic analyses reveals *Neohelicosporium* gen. nov. and five new species of helicosporous hyphomycetes from aquatic habitats. *Mycol. Prog.* **2018**, *17*, 631–646, doi:10.1007/s11557-017-1366-1.
23. Lu, Y.Z.; Boonmee, S.; Dai, D.Q.; Liu, J.K.; Hyde, K.D.; Bhat, D.J.; Ariyawansa, H.; Kang, J.C. Four new species of *Tubeufia* (Tubeufiaceae, Tubeufiales) from Thailand. *Mycol. Prog.* **2017**, *16*, 403–417, doi:10.1007/s11557-017-1280-6.
24. Yu, X.D.; Dong, W.; Bhat, D.J.; Boonmee, S.; Zhang, D.I.; Zhang, H. *Cylindroconidiis aquaticus* gen. et sp. nov., a new lineage of aquatic hyphomycetes in Sclerococcaceae (Eurotiomycetes). *Phytotaxa* **2018**, *372*, 79–07, doi:10.11646/phytotaxa.372.1.6.
25. Luo, Z.L.; Hyde, K.D.; Liu, J.K. (Jack); Maharachchikumbura, S.S.N.; Jeewon, R.; Bao, D.F.; Bhat, D.J.; Lin, C.G.; Li, W.L.; Yang, J.; et al. Freshwater Sordariomycetes. *Fungal Divers.* **2019**, *99*, 451–660, doi:10.1007/s13225-019-00438-1.
26. Hyde, K.D.; Norphanphoun, C.; Maharachchikumbura, S.S.N.; Bhat, D.J.; Jones, E.B.G.; Bundhun, D.; Chen, Y.-J.; Bao, D.-F.; Boonmee, S.; Calabon, M.S.; et al. Refined families of Sordariomycetes. *Mycosphere* **2020**, *11*, 305–1059, doi:10.5943/mycosphere/11/1/7.

27. Liu, J.K.; Yang, J.; Maharachchikumbura, S.S.N.; McKenzie, E.H.C.; Jones, E.B.G.; Hyde, K.D.; Liu, Z.Y. Novel chaetosphaeriaceous hyphomycetes from aquatic habitats. *Mycol. Prog.* **2016**, *15*, 1157–1167, doi:10.1007/s11557-016-1237-1.
28. Yang, J.; Liu, J.K.; Hyde, K.D.; Bhat, D.J.; Jones, E.B.G.; Liu, Z.Y. New species of *Sporoschisma* (Chaetosphaeriaceae) from aquatic habitats in Thailand. *Phytotaxa* **2016**, *289*, 147–157, doi:10.11646/phytotaxa.289.2.4.
29. Chuaseeharonnachai, C.; Somrithipol, S.; Suetrong, S.; Klaysuban, A.; Pornputtapong, N.; Gareth Jones, E.B.; Boonyuen, N. *Conioscypha nakagirii*, a new species from naturally submerged wood in Thailand based on morphological and molecular data. *Mycoscience* **2017**, *58*, 424–431, doi:10.1016/j.myc.2017.06.003.
30. Chuaseeharonnachai, C.; Somrithipol, S.; Boonmee, K.; Nuankaew, S.; Boonyuen, N. *Tretoheliocephala cylindrospora* sp. nov., an asexual fungus from Thailand. *Mycotaxon* **2019**, *134*, 475–480, doi:10.5248/134.475.
31. Hyde, K.D.; Chaiwan, N.; Norphanphoun, C.; Boonmee, S.; Camporesi, E.; Chethana, K.W.T.; Dayarathne, M.C.; de Silva, N.I.; Dissanayake, A.J.; Ekanayaka, A.H.; et al. Mycosphere notes 169-224. *Mycosphere* **2018**, *9*, 271–430, doi:10.5943/mycosphere/9/2/8.
32. Yang, J.; Liu, J.K.J.; Hyde, K.D.; Gareth Jones, E.B.; Luo, Z.L.; Liu, Z.Y. *Aquimonospora tratensis* gen. et sp. nov. (Diaporthomycetidae, Sordariomycetes), a new lineage from a freshwater habitat in Thailand. *Phytotaxa* **2019**, *397*, 146–158, doi:10.11646/phytotaxa.397.2.2.
33. Phookamsak, R.; Hyde, K.D.; Jeewon, R.; Bhat, D.J.; Jones, E.B.G.; Maharachchikumbura, S.S.N.; Raspé, O.; Karunarathna, S.C.; Wanasinghe, D.N.; Hongsanan, S.; et al. Fungal diversity notes 929–1035: taxonomic and phylogenetic contributions on genera and species of fungi. *Fungal Divers.* **2019**, *95*, 1–273, doi:10.1007/s13225-019-00421-w.
34. Yang, J.; Liu, J.K.; Hyde, K.D.; Jones, E.B.G.; Liu, Z.Y. Two new species in Fuscosporellaceae from freshwater habitats in Thailand. *Mycosphere* **2017**, *8*, 1893–1903, doi:10.5943/MYCOSPHERE/8/10/12.
35. Yang, H.; Dong, W.; Yu, X.D.; Bhat, D.J.; Boonmee, S.; Zhang, H. Four freshwater dematiaceous hyphomycetes in Sordariomycetes with two new species of *Parafuscosporella*. *Phytotaxa* **2020**, *441*, 19–34, doi:10.11646/PHYTOTAXA.441.1.2.
36. Li, G.J.; Hyde, K.D.; Zhao, R.L.; Hongsanan, S.; Abdel-Aziz, F.A.; Abdel-Wahab, M.A.; Alvarado, P.; Alves-Silva, G.; Ammirati, J.F.; Ariyawansa, H.A.; et al. Fungal diversity notes 253–366: taxonomic and phylogenetic contributions to fungal taxa. *Fungal Divers.* **2016**, *78*, 1–237, doi:10.1007/s13225-016-0366-9.
37. Chuaseeharonnachai, C.; Suetrong, S.; Nuankaew, S.; Somrithipol, S.; Hongsanan, S.; Srikitikulchai, P.; Jones, E.B.G.; Boonyuen, N. *Synnematotriadelphia* gen. nov. (*S. stilboidea* comb. nov. and *S. synnematofera* comb. nov.) and *Triadelphia hexaformispora* sp. nov. in the family Triadelphiaceae. *Mycol. Prog.* **2020**, *19*, 127–137, doi:10.1007/s11557-019-01547-6.
